# Electrical stimulation of chicken embryo development supports the* Inside story* scenario of human development and evolution

**DOI:** 10.1038/s41598-024-56686-y

**Published:** 2024-03-27

**Authors:** Vincent Fleury

**Affiliations:** Laboratoire Matière et Systèmes Complexes, Université de Paris-Cité, 10 Rue Alice Domont et Léonie Duquet, 75013 Paris, France

**Keywords:** Biophysics, Computational biology and bioinformatics, Developmental biology, Evolution, Materials science, Physics

## Abstract

Animal evolution is driven by random mutations at the genome level. However, it has long been suggested that there exist physical constraints which limit the set of possible outcomes. In craniate evolution, it has been observed that head features, notably in the genus *homo*, can be ordered in a morphological diagram such that, as the brain expands, the head rocks more forward, face features become less prognathous and the mouth tends to recede. One school of paleontologists suggests that this trend is wired somewhere structurally inside the anatomy, and that random modifications of genes push up or down animal forms along a pre-determined path. No actual experiment has been able to settle the dispute. I present here an experiment of electric stimulation of the head in the chicken embryo which is able to enhance the magnitude of tension forces during development. This experimental intervention causes a correlated brain shrinkage and rotatory movement of the head, congruent with tissue texture, which proves that head dilation and flexure are intimately linked. Numerical modelling explains why the brain curls when it dilates. This gives support to the idea that there exists, in the texture of the vertebrate embryo, a latent dynamic pattern for the observed paleontological trends in craniates towards *homo*, a concept known as *Inside story.*

## Introduction

Needless to say, the origin of humans is a delicate question. It is universally admitted that mutations followed by selection cause progressive changes in animal and hominids anatomy, so that the observed taxons are the result of a long Darwinian process^1,2^. However, despite the randomness of mutations, some direct trends are often observed, called orthogenesis^3^. These have received some molecular support. For example: limbs of mice can be extended in a straightforward manner, by inserting the regulatory sequence of bat cartilage genes in place of that of mice cartilage^4^. In a similar way, edition of genes of growth factors in the lamprey by CrispR-Cas9, cause elongation of the trunk as if the trunk structure were preserved and only some force cursor was pushed forward^5^. In these experiments, it is assumed that the overall physical structure is the same, and only magnitudes of molecular developmental forces are modified. Changing the magnitude of some force regulation, but not the texture, causes a deterministic developmental trend (e.g. finger or neck elongation) as if nature were playing with a structural cursor.

**Figure 1:**
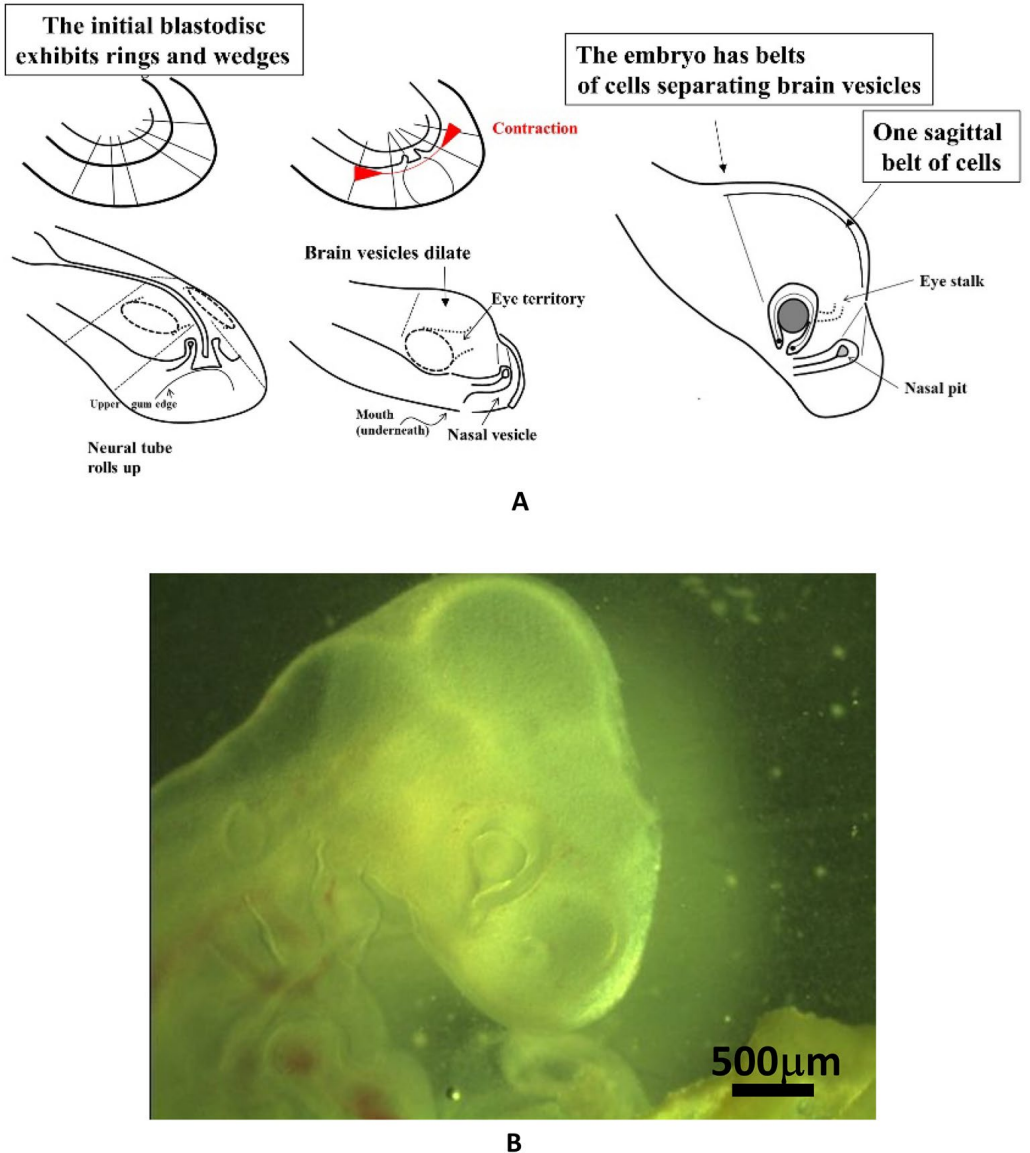
Mechanism of head formation. (**A**) Proposed scheme of head development from the blastodisc stage until the embryo stage HH18 (as hypothesized in Ref.^15^). Head formation occurs by contraction and folding of a flat blastula inside which there exists a radial and orthoradial pattern of lines (top left)^10,12^. This pattern of lines fixes important landmarks as boundaries projected on the neural tube. These boundaries form contracting rings which segment the neural tube into vesicles. The eye stalk, the brain and nasal vesicles balloon out in between the rings, because the rings hinder the ballooning (a linear contracting ring with tension **T**, exerts a centripetal force **T**/R^12,15^). In the final embryo head (to the right) there are belts of cells whose contraction generates valleys on the embryo head, plus one sagittal belt of cells which makes a sagittal valley. (**B**) A chicken head at day 3.5 of development showing a set of vesicles, separated by valleys (the valleys are visible across the surface ectoderm). The valleys correspond to belts of cells hindering the dilation of the neural tube. In between the valleys, the brain vesicles balloon out. There exists one additional valley along the median axis forming a sagittal furrow, particularly visible on the nasal vesicle (full scale 3 mm).

**Figure 2:**
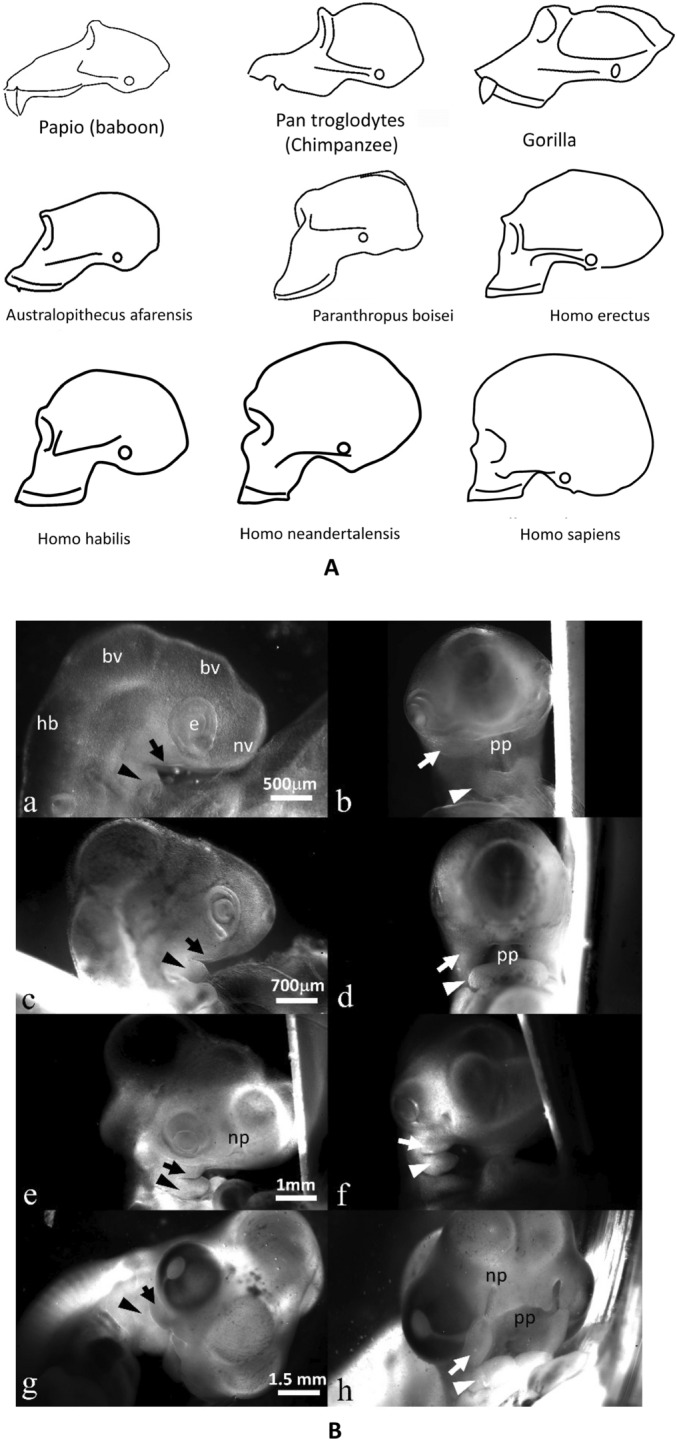
Brain and face correlation in hominins. (**A**) Profile of skulls of hominins (adapted from several public sources^19,20^). There exists an apparent recession of the face, and a correlated brain expansion and curl or rotation, recognizable in the skull pattern. It is not meant here, that baboons are ancestors of humans, but that there is a generic movement which correlates brain size, face prognathism and brain rotation. The drawings might not be exactly to scale; according to Ref.^20^ the brain volumes (in cc) are: Australopithecus 440, Paranthropus 519, Homo habilis 640, Homo erectus 930, Homo sapiens sapiens 1350, Homo sapiens neandertalensis 1400. (**B**) Dynamic origin of the upper maxillary. The images show lateral and facial views of live chicken embryos at different stages from early day 3 to day 5. The same embryos are used for the facial and lateral views. It is important to understand that during head flexure, the mouth ring (labelled upper gum edge in Fig. 1A) is flexed down as the neural tube passes over it. This ring, present on the blastodisc is projected during neurulation to become the primary palate edge (pp). The maxillary (arrows) and mandibular (arrowheads) primordia grow out from sectors of tissue located laterally. The maxillary primordia extend following precisely the mouth ring cue, so they are exactly adjusted to the primary palate ring. Hence maxillary position is slaved to head flexure. This is why maxillary recession correlates with neural tube flexure. If the neural tubes flexes more or less, the maxillary position will be less or more forward. *bv* brain vesicle, *hb* hindbrain, *nv* nasal vesicle, *e* eye; *np* nasal pit, *pp* primary palate.

The question arises, then, of what is the origin and the shape of the tissue texture in early stages of head development, and whether the texture might contain some dynamic arrow locked in, that would explain certain paleontological trends. In the last decades, it has been observed that physical stresses^6–8^ and orientation texture^9^ play a very important role in development. Recently, it has been shown that vertebrate development occurs in two steps^10^: first there is a physical cleavage of a round disk into sectors and rings, and secondly, the rings and sectors fold and form a hollow tubular animal with brain vesicles and sensory organs (Fig. 1A). A texture of oriented cell cables or strings is formed along the lines of cleavage, and the embryo folds by buckling locked along these lines^10–12^. The molecular machinery for force actuation is actin and myosin^13,14^. Brain vesicles dilate in between the cables as a consequence of closure of the neural tube^15^. This leads in 3–4 days to a typical vertebrate head as the chicken head, with brain vesicles separated by valleys (Figs. 1 and 2). While this mechanism of development has been first described in vertebrate embryos^15^, recent work has confirmed the existence of analogous cables in the formation of the drosophila head^16^.

**Figure 3:**
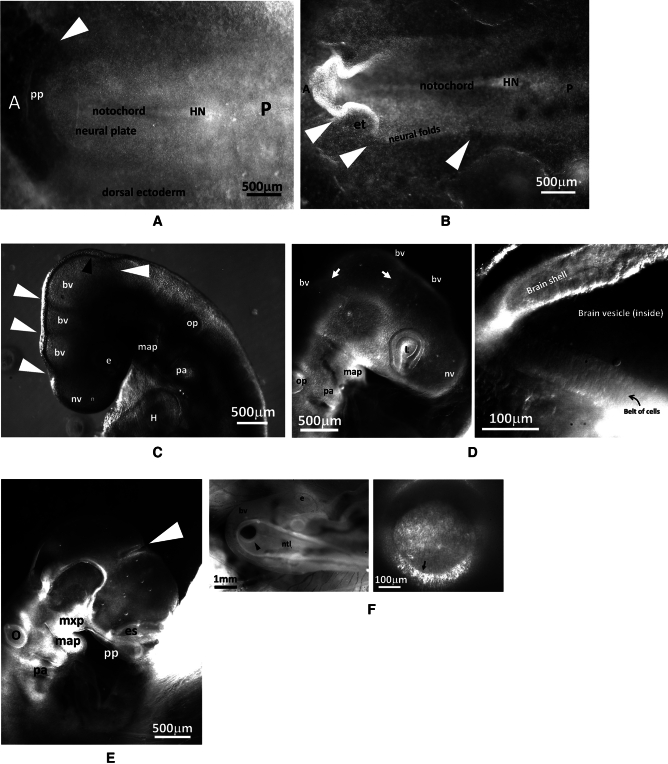
Experimental evidence of belts of cells in early chicken embryo. (**A**) After gastrulation, at the Hensen’s node recession stage (HH5, day 2, notochord is already visible along the median axis) the embryo presents a visible pattern of rings and sectors (HN: Hensen’s Node; A: Anterior; P: Posterior; pp: primary palate edge). (**B**) At neurulation stage (HH7-8, end of day 2) the lips of the neural folds show kinks in the ectoderm in front of the sector boundaries, which pre-pattern future brain segments (et: eye territory; HN: Hensen’s Node). (**C**) At an early stage (HH 18, day 3), the head exhibits brain vesicles separated by valleys (white arrowheads). There also exists a sagittal valley along the median axis (black arrowhead). These valleys correspond to belts of cells which hinder neural tube ballooning. (*bv*: brain vesicle; *e*: eye;* nv*: nasal vesicle; *op*: otic pit; *map*: mandibular primordium; *H*: Heart; *pa*: pharingeal arches). (**D**) Left, an embryo at stage HH19 observed under a Leica binocular at 2.5×, the belts of cells are visible on the midbrain vesicle (arrows) (*bv:* brain vesicle; *L:* lens; *nv:* nasal vesicle; *op:* otic pit; *map*: mandibular primordium; *pa*: pharingeal arches). (**D**) Right Direct white light observation of the location of intervesicle valley along the ventral side, inside the midbrain vesicle, after splitting the embryo along the sagittal direction with micro-scissors, same embryo as (**D**) Left, under a Nikon microscope at 20×; the belts of cells appear more clearly from the inside of the embryo. The width of the belt of cells at this stage is *ca.*90 µm, as seen on the floor of the neural tube. (**E**) On the dorsal side, the valley (arrowhead) between vesicles is as deep as 2/3 of the neural tube lumen (*es*: eye stalk; *o*: otic pit; mxp: maxillary primordium; *map*: mandibular primordium; *pp*: primary palate; *pa*: pharyngeal arches). (**F**) An embryo head at day 3 HH18, observed from the dorsal side (which is more transparent). It is possible to image the inside of the embryo head across the neural ectoderm and see directly the cell collar (arrowhead) which separates the hindbrain vesicle and the midbrain vesicle. Direct imaging shows that the cells stack and register around the ring (arrow), forming a collar or belt of cells organized in a radial order (the long axis of cells is oriented radially). *ntl*: neural tube lumen; *e*: eye; *bv:* brain vesicle.

Now, turning to paleontological studies, it has been suggested that human skulls can be organized in a deterministic order by which the brain is bigger, the head and neck are flexed more forward, and the face is progressively less prognathous^17,18^ (Fig. 2A). This handful of correlated anatomical facts might explain the trend between pre-hominians and hominids. Nose position and maxillary (upper jaw) recession are naturally correlated to head flexure (Fig. 2B) because the edge of the palate comes from one anterior ring of the blastodisc (Figs. 1A and 3A). Such internal correlations were well known of Charles Darwin which mentions in his book “the mysterious law of correlation of growth”^21^. At a technical level, such correlations might be explained by flowing the embryonic tissue deterministically more or less forward in a visco-elastic movement during development^22,23^. The physical field correlates the effects at long distance.

Now, I report here an experiment of electrical stimulation of head development in the chicken embryo at 3 days of development or developmental stages HH 18–20, following the nomenclature of Hamilton and Hamburger^24^. It has long been known empirically that electric stimulation of tissues tends to generate organized contractions which follow tissue texture^25^. More recently we have shown that, during embryo development, electric stimulation enhances contraction forces by a factor between 2 and 10 (Ref.^26^). The pattern of contraction induced by electric stimulation follows the texture, and it causes deformations which are congruent with the texture, both in magnitude and in orientation. This to say that the contraction movements propagate anisotropically following the tissue texture^26^. When increasing suddenly the tension stress holistically in a developing embryo, a deterministic morphogenetic effect is obtained, which is not obvious. Here we stimulate the embryo head electrically, and observe the resulting movements. The electric stimulation which is reported here reveals an internal correlation between head flexure and dilation, related to the texture of the embryonic tissue and of the vessels within it.

To complete the work, I present a numerical simulation of brain expansion which clarifies the important role of the number of brain vesicles and of the number of cellular collars in between them, and explains why texture constrains the dynamics of brain expansion, and how dilation and flexure are correlated.

## Results

It is classically observed that during normal craniate embryo development, the embryo head rocks forward and the mouth folds inwardly (Video 1, 15 h). As development continues, the brain and nasal vesicles balloon out^15^. Their development is hindered along the cables described in Ref.^15^, these cell alignments are quite visible, even in the absence of any staining (Fig. 3, esp. Figure 3D,F). On the dorsal side, at stage HH19, the valleys between vesicles may encompass up to two thirds of the neural tube lumen (Fig. 3E), they strangle the tube so that vesicles balloon out between the valleys like hernias. When an electric shock is given two different effects may be obtained.

### Results: retrograde rotation and brain contraction

**Figure 4:**
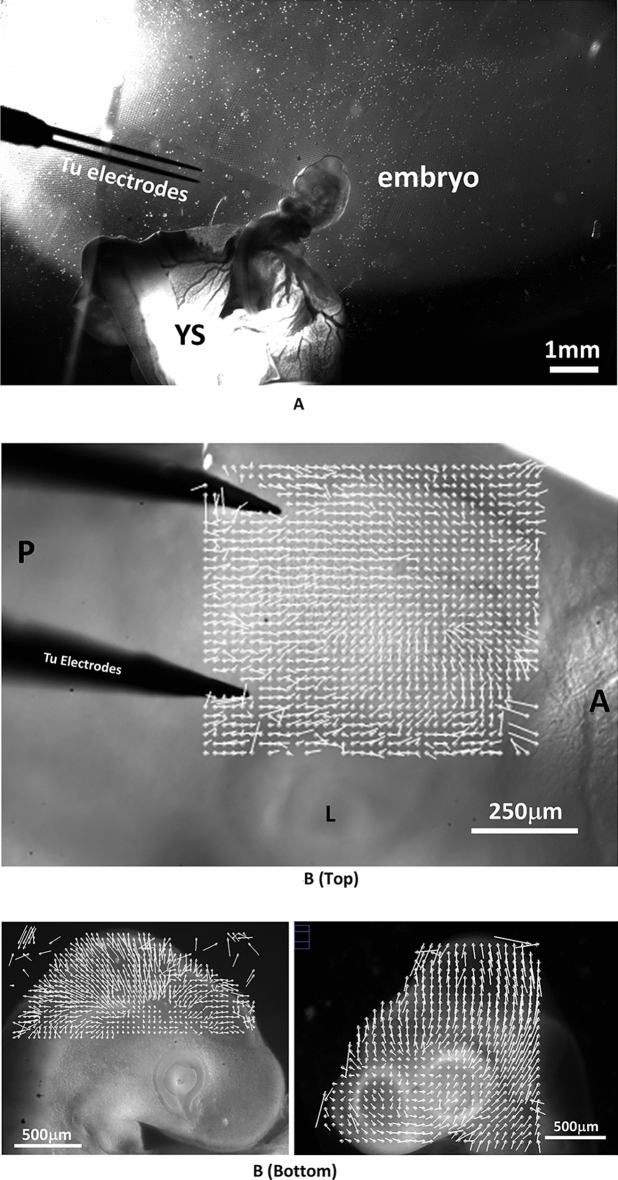

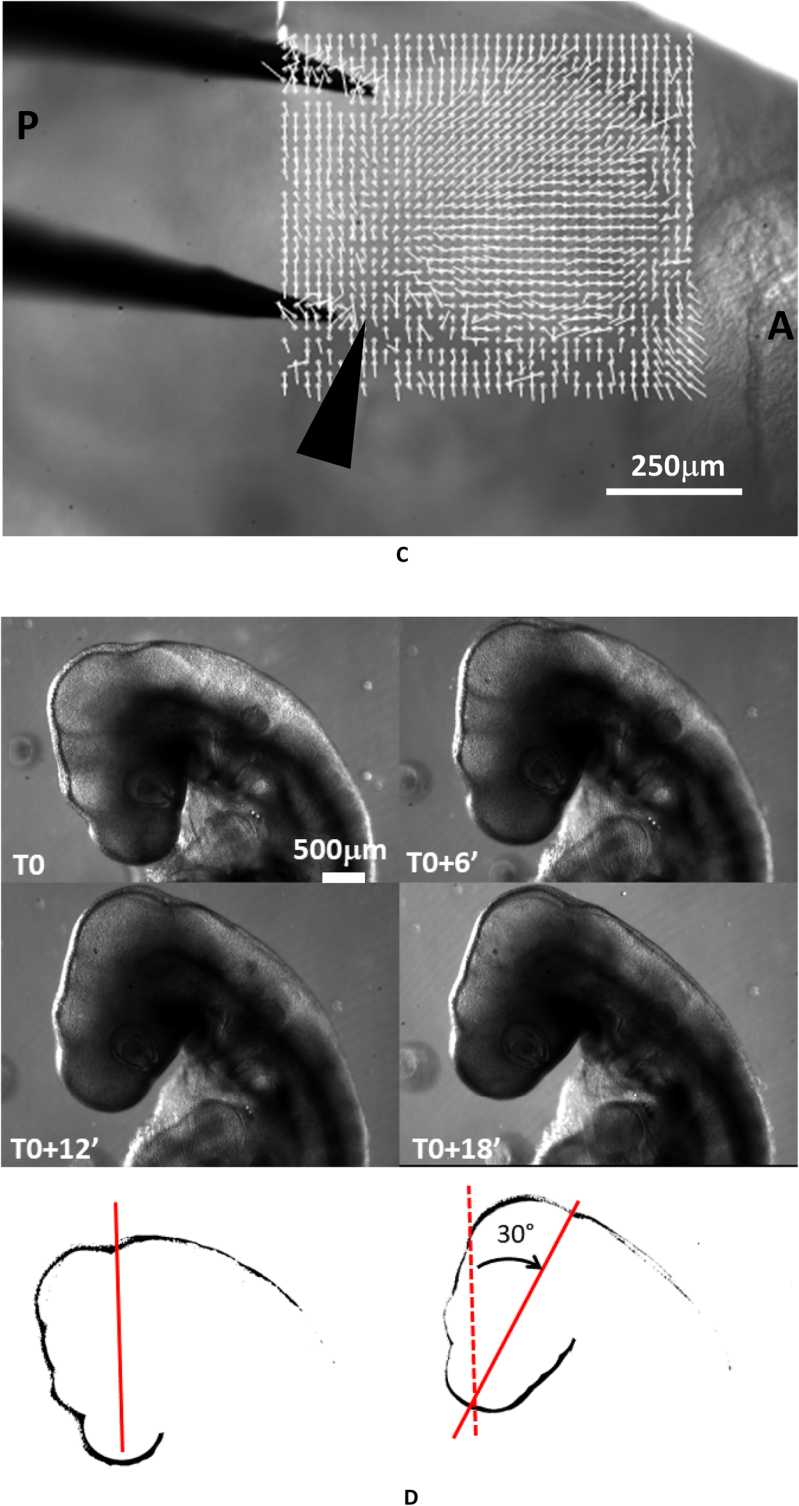

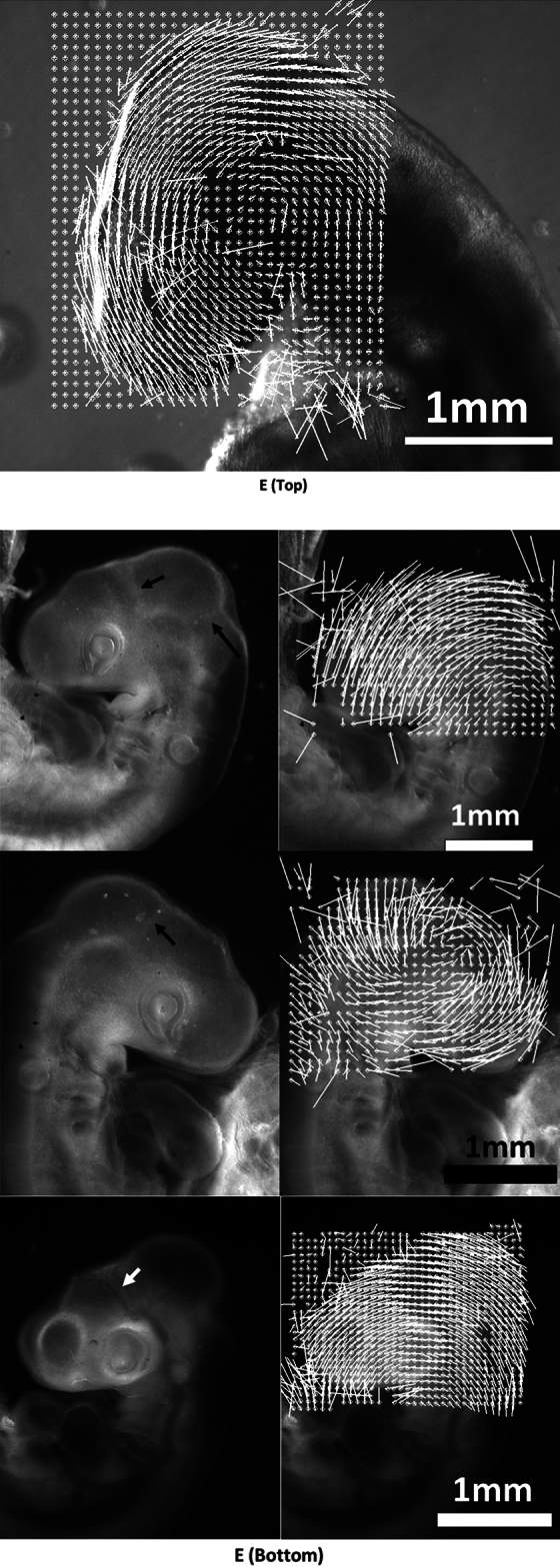
Effect of electrical stimulation at developmental stage HH 20. (**A**) shows the situation of the electrodes approaching the embryo (Mag. 0.7×, day 3; *YS*: Yolk Sac). (**B**) Top shows a dorsal view at mag. 10×. The electrical stimulation (0,1 V, 1 s, day 3) causes a shrinkage of the brain vesicle. We measure the contraction pattern by the PIV method in ImageJ, which gives the displacements; here the displacements are calculated from a top (dorsal) view between plates separated by 3 min and the movements are scaled to visible sizes (A = Anterior, P = Posterior). The presence of a centripetal pattern of movement evidences a contraction of the brain vesicle, which is directly visible on the videos. The eye is visible in the lower part of the image (*L*: Lens, from Video 2). (**B**) Bottom Left lateral view at mag. 2.5×, after correction of the head rotation with the Stackreg tool in ImageJ, of the displacement map following an electric stimulation in a HH19 embryo (day 3). It shows a contraction in the area of the brain (interval between frames for the PIV map 5 min, same embryo as Fig. 3E in which the rotatory component is shown). (**B**) Bottom Right, brain contraction following electric stimulation in a stage HH20 embryo (day 4); same embryo as one in Fig. 3E in which the rotatory component is shown. (**C**) The electrical stimulation causes also a contraction of the belt of cells separating the brain vesicles, which causes a retrograde movement of flexure. The PIV map shows the contraction along the boundary between brain vesicles (arrowhead), characterized by convergence of movements towards the valley. The gradient of movement evidences the presence of a shear force oriented posteriorly. Surprisingly, the brain contraction and the belt contraction are slightly offset in time, which allows one to separate the two effects (we ascribe this delay to propagation of the stimulus). (**D**) The electrical stimulation causes a retrograde rotation of the entire neck (analyzed here by contour extraction in ImageJ), as if the physiological movement of head and neck flexure was reverted. The speed of rotation in this assay is 1.5°/min (see Video 3). The effect lasts for 20 min approximately, after which the head rocks back in the physiological direction, albeit with a temporal delay (see Video 3). The maximum effect obtained is 30° of retrograde flexure. (**E**) Top When analyzed by PIV, the movement of the head exhibits an organized retrograde rotation, as if the head was mounted on a hinge (the data corresponds to (**D**), Video 3; (**E**) Bottom: three other embryos at slightly different times (day 3 and day 4); PIV map calculated at 5 min. interval, (arrows point to visible cell belts). The movement is calculated by generating a grid of virtual points (crosses) on the pattern, and then calculating the movement from these points by correlation function (PIV) between two plates (Macros available upon request). The length of the vectors is scaled to visible sizes. The pattern of vector gives the instantaneous displacement field or “flow field” of tissue during these 5 min, in almost all cases (16/19) we evidenced a rotatory movement of the tissue. Video 4 shows an animation based on two stages of electrical stimulation showing sharply the brain shrinkage accompanying the head rotation.

For moderate values of the electric stimulation, (0.02–0.1 V, 1 s, Fig. 4A), a set of correlated events is observed (N = 19; 16 positive/19). (1) The brain vesicles shrink (Fig. 4B, Video 2). (2) The belts of cells hindering development constrict (Fig. 4C, Video 2) (3) The entire head rocks (Videos 3 and 4), or rotates in a pattern of rotation which is retrograde to the physiological movement: the nasal features become more anterior, the neck flexes backwards, and the mouth primordia become more anterior (Fig. 4D), by up to 30° in 20 min.

When the pattern of movement is analyzed laterally by Particle Imaging Velocimetry (PIV), the movement is seen to be quite organized: it forms a rotation, almost like a hinge, around a point roughly centered on the mouth corner (Fig. 4E Top). Figure 4E Bottom shows three other examples of the effect of electric stimulation on an embryonic head, including one at a later stage (day 4). The head rotation is quite conspicuous.

From a temporal point of view, the movements which are observed show a rotation speed of up to 1.5°/min, and a brain deformation rate of 0.02/min. Nevertheless, the movement induced by the electrodes progressively slows down, and after 20–60 min, the movement may return to a physiological orientation (the flexure of the head is reversed again, see end of Video 3).

**Figure 5:**
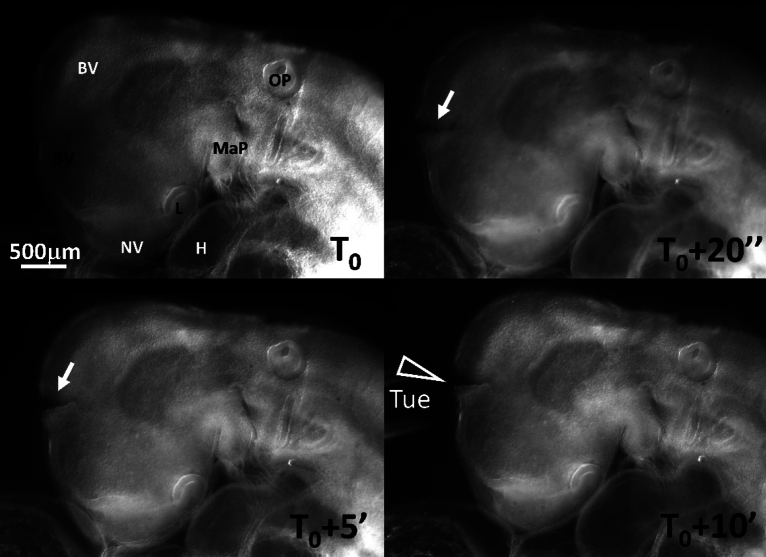
Evidence of tension in the brain shell. In order to demonstrate that there is tension in the brain shell, and that electric stimulation enhances the tension, we first do an incision in one brain vesicle (Top Right, arrow), with a scalpel. The wound is left to open for 5 min (Bottom Left). Then, the electrodes are approached and an electric stimulation of 0.15 V is applied in a pulse of duration 1 s. The wound is followed by time-lapse (Video 5). It shows a widening of the cleft, and a more pronounced concave bending profile after the electric stimulation (arrowhead). The concave profile of the lip of the wound is a consequence of stress equilibrium at the wound surface. The stimulated wound does not heal, while without electric stimulation, the wound rapidly heals (Video 6). *BV* brain vesicle; *NV:* nasal vesicle; *MaP:* mandibular primordium; *OP:* otic pit; *L*: lens; *H* heart; *Tue:* tungsten electrodes.

In order to evidence the presence of tension in the brain shell, I incised the brain of embryos with a sharp straight scalpel (Swann Morton Ref. 0563) (N = 3). The brain shell opens rapidly (10 s) and forms a cleft, with a concave shape of the lips, as classical in wound formation around a stressed tissue (Video 5, Fig. 5 Top Right, and Bottom Left, the data acquisition rate is 1 frame/10 s). The concave form of the lip of the wound is due to the gradient of stress at the lip which generates a sideways force (by continuity with the surrounding fluid, the stress is zero at the surface of the wound, and tensile inside; see for example similar experiments on the amniotic sac in Ref.^27^, see also mechanical analogs in Supp. Fig. 6). However, in order to prove that electric stimulation enhances the said tension, I managed to approach the electrodes with one polarity on each side of the wound (great care must be taken during positioning and orientation of the embryo under the microscope for this dual incision + electric stimulation experiment to be possible). In this experiment, the incision is first performed without electric stimulation, and after 5 min during which the cleft is left to open by itself, an electric stimulation is given. By passing a stimulation of 0.15 V during 1 s across the already open wound, it is observed that the wound opens more, and the surface concave feature, which witnesses the presence of a tension, becomes more marked in a few minutes (Video 5, Fig. 5 Bottom Right). This shows that the range of potential used indeed has a contractile effect and, as importantly, that the effect has the magnitude and the temporal scale of variation required to explain the phenomena which are reported here. Also, the incision shows that the tension is not just passive, there is an active pull by the tissue. While the electric stimulation shows that the tension response is active, this fact is in the first place visible in controls: indeed, after opening of the cleft by incision, if the cleft movement is followed without electric stimulation it is observed that the cleft movement is progressively reversed as cells realign along the wound and form an elliptic ring which constricts and the cleft progressively closes and heals in 1 h (Video 6, performed N = 4). This is identical to what was observed in experiments of amniotic sac incision at the same developmental stage^27^. It also shows that the tension force which serves morphogenesis can also serve wound healing.

The range of potential difference ΔV between electrodes in which the effect of electric stimulation was explored is from 0.01 up to 0.5 V. Retrograde movements were observed for 0.02 < ΔV < 0.2 V. For lower values of ΔV, the embryo exhibits only a very short contraction twitch which relaxes quite rapidly (data not shown). It was noted that the distance between electrodes (250 µm or 500 µm) was a relevant issue. The experiments reported here are for 500 µm of electrodes interdistance. The rationale for the magnitude of the effect is not obvious because tissue reaction depends on the developmental stage of the embryo. It is likely that the visco-elastic properties, the magnitude of the available forces, and the thresholds for excitation change during development.

### Results: anterograde rotation and brain dilation

**Figure 6:**
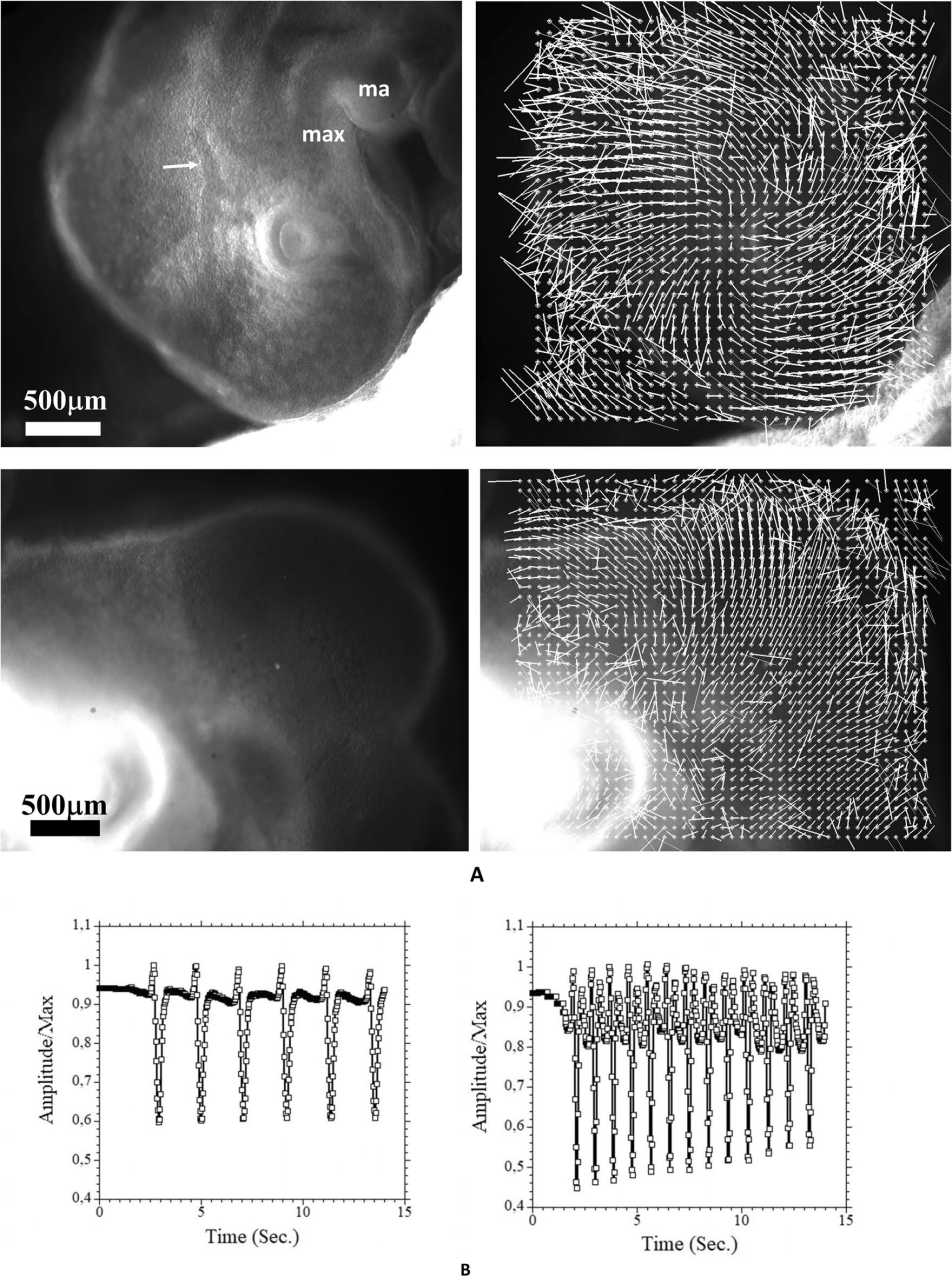

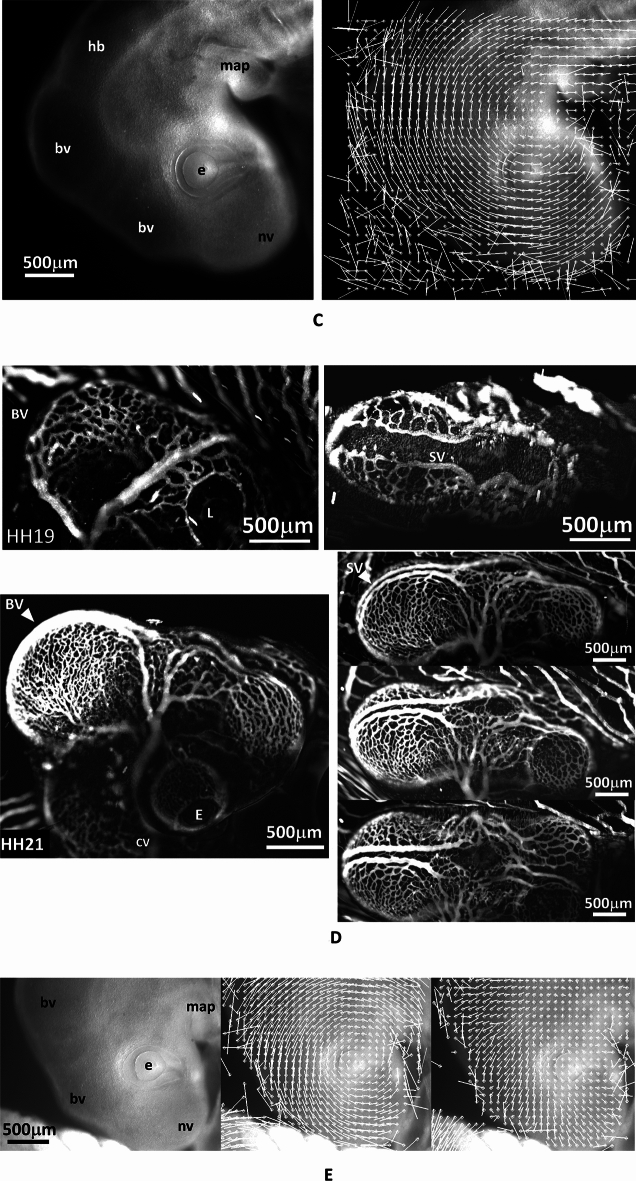
Positive rotation induced by electric stimulation. (**A**) Electric stimulation may also induce a brain dilation associated to anterograde flexure. The plate shows two examples, with electric stimulation 0.2 V and 0.15 V (Videos 8 and 9). In Video 9 both the anterograde movement and the retrograde movement can be observed, and the concomitant variation of diameter of the cephalic vein (duration of the Videos 55 and 50 min.). Video 10 concatenates 2 frames during brain expansion to show the simultaneous expansion and flexure, in Video 10 the arrowhead points to the dilating cephalic vein. *bv*: brain vesicle; *ma*: mandibular primordium; *max*: maxillary primordium; *hb*: hindbrain; *e*: eye. Arrow points to the enlarged vessel. (**B**) The graphs show the heartbeat as monitored by Video Cardiography (see Materials and methods, and Supp. Fig. 7), before and 5 min. after an electric stimulation. The video cardiography follows in vivo the movement of the heart wall. The measured data is proportional to the heart wall displacement speed. A strong electric stimulation (0.15 V) induces an increase in frequency and heart thrust which causes an increase in blood flow and pressure, which correlates with the rapid brain expansion. Heart rate increase correlates with the dilation of the blood vessels (arrow in (**A**), see also Video 7). (**C**) After 1–2 h, the heart rate returns to normal and the head undergoes a contraction and a retrograde rotation (Video 11, 3 other examples available). *bv*: brain vesicle; *nv*: nasal vesicle; e: eye; *map*: mandibular primordium. (**D**) Vascular structure at stage HH19 (Day 3), and HH21 (Day 4). *BV*: brain vesicle; *SV:* sagittal vessels; *CV:* cardinal vein; *E:* eye. The blood vessels exhibit a texture which mirrors the underlying embryo texture. The brain vesicles are covered by a plexus of small capillaries, while larger vessels follow the intervesicle valleys and the longitudinal body axis. The three images to the right in HH21 show images of blood vessels in the same embryo at same time and different sagittal oblique views, obtained by gently rocking the embryo in vivo with a motorized precision stage (see also Supp. Fig. 8 for the flow directions). (**E**) If we simply poke the omphalomesenteric artery on one side of the embryo, we provoke a bleeding which reduces the blood pressure. This causes, without any electric stimulation, a retrograde movement and a brain shrinkage (experiment performed N = 5, 5 positive/5). Here we show the rotatory movement of the head (Middle) and the shrinkage of the brain vesicule (Right), after digital removal of the rotatory component of the movement with the Stack-Reg plugin in ImageJ. These data correspond to Video 12 (the duration of this Video is 25 min, the data for the (**E**) are obtained between frames separated by 80 s). *bv*: brain vesicle; *nv:* nasal vesicle; *e*: eye; *map:* mandibular primodium.

However, for higher values of the voltage > 0.2 V, we observed a transient contraction (~ 5 min) followed by an overshoot during which embryo development accelerates. In this case the blood vessels show a conspicuous dilation (Videos 7–9, Observed N = 8), and the head shows a dilation, accompanied by a correlated forward flexure (Fig. 6A, Videos 8 and 9). Video 10 shows a concatenation of two frames at 20 min. interval showing the anterograde flexure, accompanied by a dilation of the brain vesicles. This is strictly the opposite effect as the one shown in Video 4. When studying in more detail the origin of this phenomenon, it is seen that it correlates with an increase in heart rate, caused by the electric stimulation (Fig. 6B), which is the obvious cause of vascular dilation. This shows that variations in blood flow and pressure may affect the rate of brain dilation, but the correlation between dilation and flexure is “built in”. Indeed, when the heart calms down (heart rate is monitored visually by video microscopy), after about 2 h, the head constricts again and the flexure reverts to retrograde correlatively (Fig. 6C, Video 11, please note that in this Video, the embryo head is first dilating, and next contracting, see also Video 9 in which the anterograde and retrograde movements are both quite visible; N = 5). The increase in pressure propagates through a vascular bed which exhibits an organized pattern quite adapted to the embryo form (Fig. 6D): larger vessels pass through the valleys, and smaller vessels span the brain vesicles in a fanning pattern of threads (there exist also sagittal vessels).

One can hypothesize that, if indeed the increase in blood pressure is the cause of the overshoot of brain dilation (accompanied by an anterograde movement of rotation), then, decreasing blood flow in the normal physiological situation (without applying any electric stimulation) would cause a retrograde rotation (accompanied by a brain shrinkage). Also, one might think that the retrograde movement observed after the electric stimulation is simply the return to elastic equilibrium of the movement obtained by the stimulation, and that such correlation between heartbeat and head rotation is not physiological. This is why I performed an additional control consisting in poking one omphalomesenteric artery of a “not stimulated” normal embryo with the tip of fine dissection tweezers. In this case, a slow bleeding occurs, and the blood pressure progressively decreases. As predicted, one obtains a very clear contraction of the brain accompanied by a retrograde rotation of the head (Video 12, duration 25 min), Fig. 6E (experiment performed N = 5), in the absence of any electric stimulation, by simply letting the embryo bleed. In Fig. 6E, I show the rotatory movement as measured by PIV (Middle), and the brain shrinkage after digital removal of the rotation (Right). While the movements are obvious in the Video 12 (4 other available), the separation of the rotatory movement and of the shrinkage allows one to confirm the visual impression. This control confirms that increase (resp. decrease) in blood pressure, causes also anterograde (resp. retrograde) rotation of the head.

## Modelling

**Figure 7:**
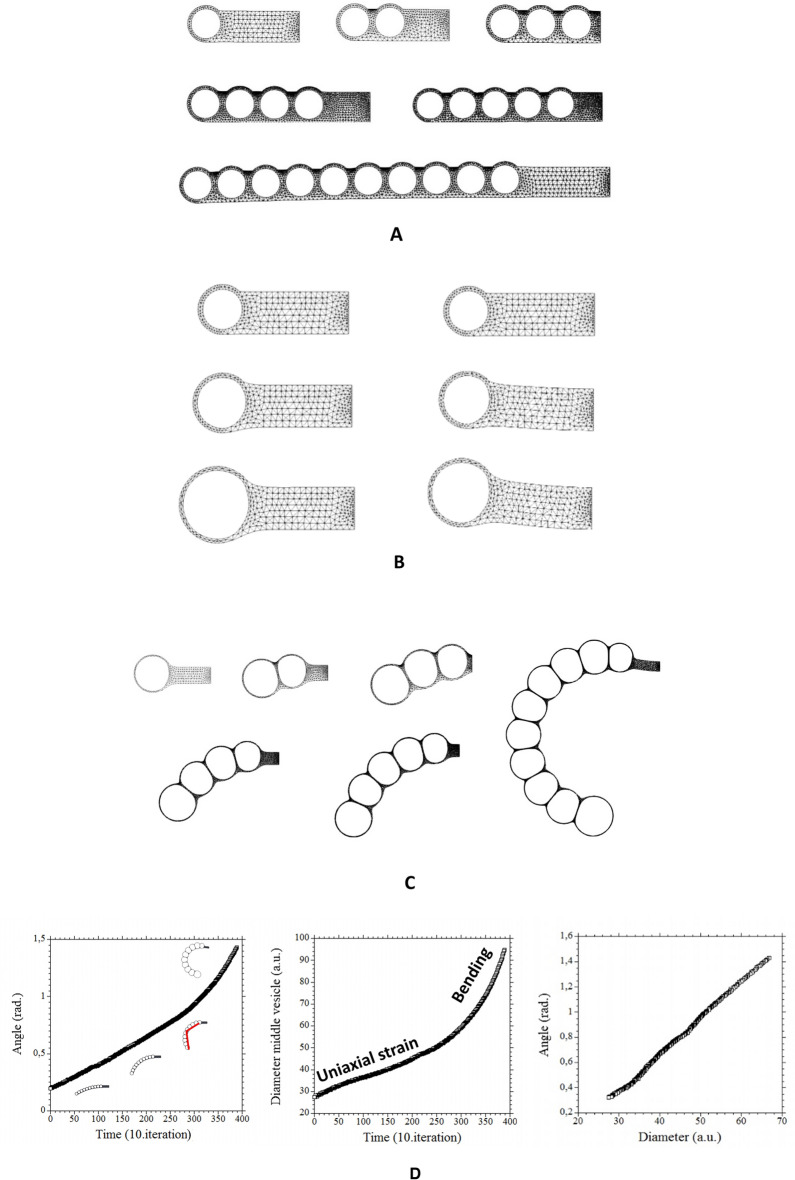
Modelling of brain vesicle expansion. (**A**) We model the head, just after neurulation, as a series of vesicles. For the sake of the modelling we consider the cases 1 vesicle to 10 vesicles, although the actual number is 3 vesicles (4 counting the nasal vesicle as a brain vesicle), this will allow us to understand what is at play in this process. We also assume a uniform size of the vesicles at start, although the brain vesicles are not so uniformly distributed at start. We assume that there exist belts of cells in between vesicles hindering the ballooning (including one sagittal belt). There exists a gradient of contraction which results in a localized shear force oriented posteriorly inside the belts of cells. (**B**) Left When treating a single vesicle, we observe that the vesicle naturally dilates with no tendency to bend forward (see Video 12). (**B**) Right When we introduce an increase in tension, we observe a reduction of the brain vesicle, and an uplift of the head due to the shear force (see Video 13). (**C**) When performing the same simulation with several vesicles, new effects are observed: in the absence of tension in the belts, the brain vesicles dilate, and the entire “head” or neural tube flexes forward, because the dilating vesicles in series act as a linear compressive stress exerted along a linear visco-elastic object. As the number of vesicles increases a roll-up or curling can be obtained, the region between vesicles works as a hinge, and the angle increments add up. However, it is observed that the dilation of the vesicles decreases posteriorly (to the right in the simulation). The vesicle located frontally is more dilated while the posterior vesicle is less dilated (see Video 14, 4 vesicles case). It is also observed that a negative curl appears in the area of the “neck” for more curled samples such as the 10 vesicles sample, this negative curl is observed physiologically in Video 1. (**D**) When the movement is analyzed quantitatively (here the behavior of the 10 vesicles case, Video 15), it is observed that there exists an acceleration of the phenomenon, with two regimes: a linear regime at small bending, and a rapid non-linear regime at higher bending. Both the flexure (Left, in red the measured angle) and the vesicles diameter (Middle) accelerate. When the angle of flexure is plotted as a function of vesicle diameter a straight correlation is obtained (Right).

In order to describe the effects observed here, I implemented a 2D finite elements simulation (with Freefem++^28^). The hollow neural tube with the early 3 vesicles and the valleys separating them is modelled by a tubular brain, fixed at one end (the neck, to the right). We represent the early vesicles of the head by cavities. In the 3D case, there are rings hindering the ballooning (see Supp. Fig. 9). The tension in the rings causes an inwards force. To reproduce this effect, we separate the vesicles by columns, so that the internal pressure has to fight the tension in the column. In the real neural tube the vesicles communicate through the lumen. From a dynamic point of view, the pressure equilibrium between vesicles in our model, associated to the flatness of the column, implies equality of pressures, as if the lumen were open. Also, the walls in the real valleys between vesicles encompass 2/3 of the lumen diameter, so that the mechanical contact is in order of magnitude close to a full contact across the lumen. With this model, we treat the case of one, two, three, four, five and ten vesicles (Fig. 7A). The effect of pressure is modelled by a constant pressure exerted at the internal boundary of the thin hollow shells. The gradient of stress between the belt of cells in the valleys (especially the sagittal valley) and the stress in the thin hollow vesicles creates a shear force tangent to the external surface and oriented towards the neck. The code solves iteratively the deformation of the neural tube, step-by-step, in a quasi-static, over-damped, visco-elastic approach^12^. For the calculation of the movements, the embryonic material follows a constitutive equation of the form [**σ**] = [**H**]∂_t_[**ε**] where [**σ**] is the stress tensor, [**H**] the viscosity matrix (formally analogous to a Hooke tensor), and ∂_t_[**ε**] is the tensor of deformation rates. In physics, simple incompressible fluids have a single shear viscosity µ, relating shear stress σ_ij_ to shear rate σ_ij_ = µ(∂_i_**V**_**j**_), µ is an off diagonal term in [**H**]. Generally speaking, homogeneous fluids, such as Newtonian fluids, rarely have other terms in the viscosity matrix. It is assumed that the fluids flow under shear (the off-diagonal terms of the stress tensor), but that they do not shrink or expand under pressure stress (the diagonal terms). This is not so with living material, which flows under shear, but can also expand or shrink, under compressive or tensional stress. In this sense living material is a compressible fluid. This is why a so-called dilational viscosity, generally noted η, must be introduced in [**H**] such that σ_ii_ = η(∂_i_**V**_i_), as done here. We assume a very viscous behavior, such that inertia terms are neglected. The displacement rates are obtained quasi-statically, by solving numerically the equilibrium equation for [σ] and [**ε**] in the bulk with given forces at the boundaries. The deformed configuration is updated incrementally and used to calculate the new displacement rate field in a Lagrangian specification. One may think that in linear elasticity, it is the same thing to perform *n* incremental steps with a force term F, or to calculate the pattern in a single step with a force term *n* times greater. This is not so because as the pattern expands, the force term is applied to a moving boundary which changes with time, so the force term changes self-consistently with the form, and it does not simply increase linearly. In our model, the force term inside the vesicles is a uniform pressure term applied at the internal boundary of each vesicle. When introducing an additional tension T of the shell in the problem, in order to describe the situation when an electric shock is given, we take into account two effects. One is the effect on the dilating shell. Due to the curvature of the shells, the effect on each dilating shell of an increase in tension amounts to a negative pressure term oriented internally and with a formal expression κT, where κ is the curvature of the shell. This requires calculating point by point the curvature of the shell. This is not quite usual in finite elements theory, and we are grateful to Charles Dapogny for providing the curvature module for the calculation, point by point at each vertex of the boundary, of the surface curvature (the curvature module for the Freefem software is available by the author). However, we assume that there exists a linear wire inside the sagittal valley oriented from the head to the neck. When tension is increased brutally in such a mechanical system (think of a spring which is stretched brutally), there is an additional force term due to the tension gradient between the clamped end (to the right in the calculation, considered as the neck), and the free end, to the left in the calculation (considered as the extremity of the neural tube where the neural folds fold against each other at the free end, in the nose area^15^). As the wire experiences a raise in tension, the free end experiences a vanishing tension, while the clamped end experiences a maximal tension (sustained by the principle of action and reaction, in the clamping material). This creates a gradient of stress oriented from the apex of the head towards the neck, which results in a force in the sagittal valley. Stated otherwise, in this model, in which the “head” is clamped on the neck, the body can pull the head, but the head cannot pull the body. The force is implemented in the boundary condition for the finite elements calculation as a surface shear oriented from the apex towards the neck (the source code is available upon request). For the sake of simplicity, we assume a uniform surface stress gradient. Please note that we do not introduce a gradient of longitudinal tension along the vertical column separating the vesicles, because the tension in the foramen of the real system loops onto itself, while the sagittal valley stalemates at the frontal notopore^15^. However, the increase in tension itself in the vertical column (not the gradient) is implicitly present in the value of T, by continuity of the stress between the columns and the surface shell of the brain vesicles. Also, the increase in tension of the wires in the valleys, in the direction perpendicular to the valleys may create an additional tension gradient, which, in a 2D simulation simply overlaps geometrically with the gradient of tension in the sagittal valley (in a 2D profile view, the longitudinal sagittal valley collapses with the perpendicular direction of the inter-vesicle valleys), and may create a localized increase in shear force in the valleys themselves (that we neglect in this simulation).

We first let the brain with one vesicle only expand (Video 13). We obtain a vesicle which dilates (Fig. 7B Left). We then increase the tension, and obtain a smaller brain, and a dorsal flexure (Fig. 7B Right, Video 14). Now, we perform the same calculation with more than one vesicle. We obtain a very clear trend: the vesicles in series functions as dilating elements which, by expanding the tube along the A–P axis, tend to flex it down (Fig. 7C equal times), a fact which was absent with a single vesicle (see for example Video 15 with 4 vesicles). This is why as the vesicles are larger, and as the number of vesicles increases, the “head” in our simulation is more flexed. Quantitative analyses of the movement, for example the 10 vesicles case (Fig. 7D, Video 16), shows an acceleration of the phenomenon with a shift from a linear regime to a non-linear regime, which is due to a transition from uniaxial strain to bending. Indeed, as long as the neural tube is not or only slightly bent, the constant stress elongates it steadily (no feedback); however, when the tube is bent, the torque increases with bending, which creates a positive feedback and a non-linear acceleration. Now, we plot the flexure as a function of vesicle diameter (Fig. 7D Right) and get an allometric relation (in which time is out). To the bottom left of the chart we find predicted animals with a very small straight tubular brain, at the top right animals with a very large and curled brain.

**Figure 8:**
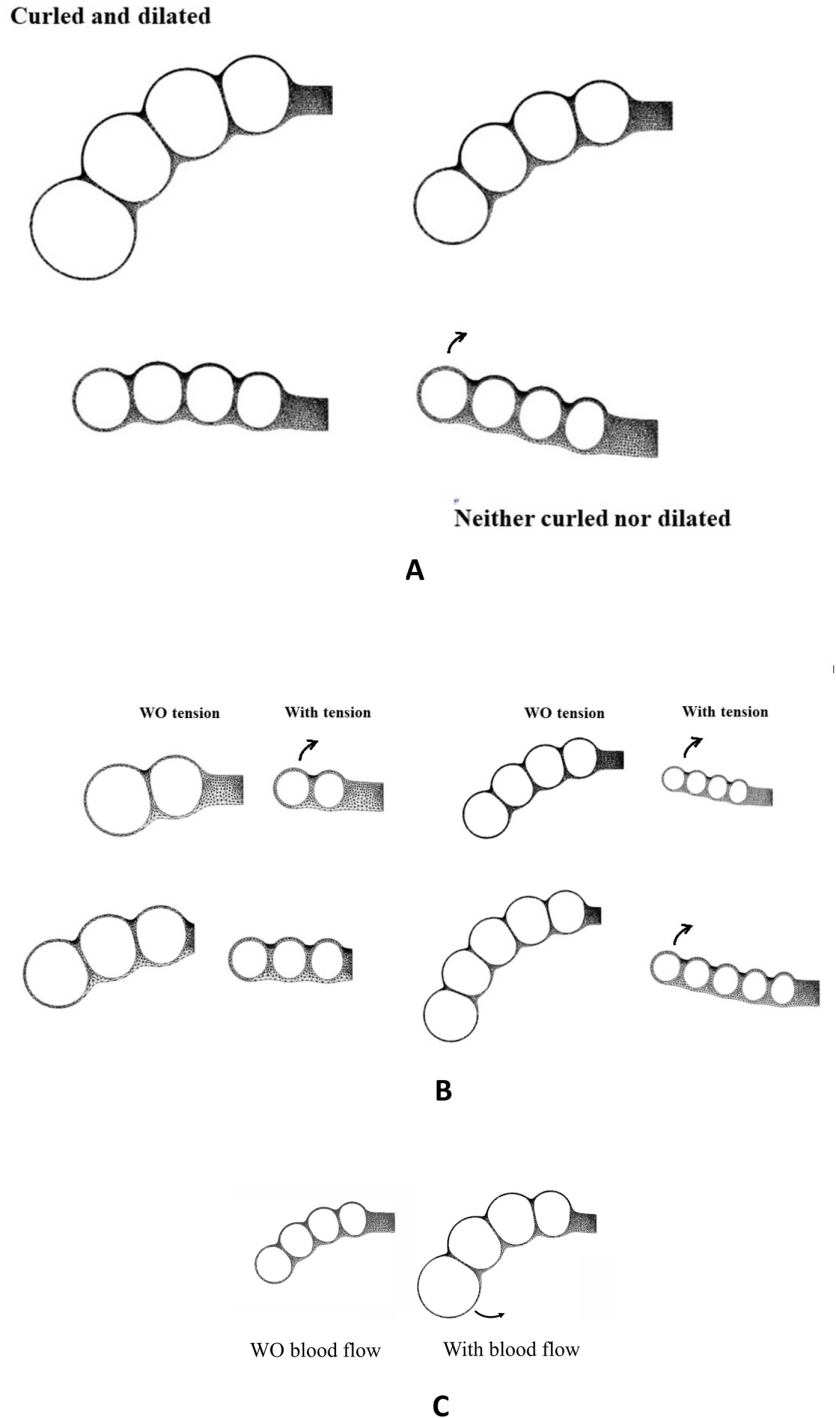
Effect of changing stresses in the model (**A**, Video 16). Tension increases from top left to bottom right; the figure shows for the case of 4 vesicles and a stepwise increasing tension (with resulting pressure inside the brain varying as (1, 1–0.02κ, 1–0.04κ, 1–0.06κ, 1–0.08κ, where κ (kappa) is the curvature, and P = R = 1 in dimensionless units), the progressive uplift of the chain of vesicles, shown at the same simulation time. (**B**) shows, at the same simulation time, the 2 to 5 vesicles development, without tension to the left, and with tension to the right. Here, with the chosen values of the parameters, the line of vesicles is straightened and uplifted, there is no more forward bending (see Video 16). (**C**) shows, at the same simulation time, the modelling of the 4 vesicles development case, without blood flow to the left, and with a blood flow to the right. While electric shock implies a higher tension in the shell and a posterior shear exerted above (causing brain shrinkage and retrograde bending), we assume that increased blood flow increases pressure in the distal capillaries shell, but it also increases the circumferential stress force in the larger blood vessels, this will appear in the model as a decreased tension in the shell (analogous to turgor dilation) and a posterior shear stress created by increased tension exerted below the neural tube, causing brain dilation and anterograde bending. The plexus over the brain vesicles behaves as a cover of strands whose tension hinders brain vesicle dilation. Increased pressure in the strands relieves the tensile stress and favors brain vesicle dilation; this effect amounts to a modified effective brain pressure of the form 1 + 0.02κ, in this simulation. The distribution of stresses for the simulations is also explained in Supp. Fig. 9.

We now add tension (Fig. 8). The belts in between vesicles function as stiffening elements which tend to straighten and uplift the head (Figs. 8A, see Video 17). There is a competition between two effects: dilation tends to rock the head forward, while belt stiffening tends to uplift the head. The interplay is not intuitive, and it depends on the number of vesicles and the magnitude of the tension in the belts (interestingly, the uplift is weaker for 3 vesicles). In all cases, an increase in tension causes smaller vesicles, associated with a less flexed head, and vice-versa (Fig. 8B).

Finally, in order to model the effect of increased blood flow, we assume, considering the shape of the vascular network (Fig. 6D, and Supp. Figs. 8 and 9) that the capillary plexus behaves as cover or cap over the brain vesicles which resists expansion like a loose fabric. When pressure is increased in the capillary bed, its tensile stress is diminished and the brain vesicle expands more (capillaries have no active tensile reflex response to vasodilation). This introduces a normal term of the form + κT in the effective pressure of the brain shell, by Laplace law. Simultaneously, increased stress in the vessel walls imparts a posterior shear stress positioned along the lower boundary of the neural tube because the vessels too follow the texture of the tissue (see stars in Supp. Fig. 8). We therefore add a shear force oriented in the posterior direction along the lower boundary in our model. In summary, electric stimulation of tissues causes a dorsal shear and a negative pressure on the brain, while electric stimulation of the heart causes a ventral shear and a positive pressure on the brain. We find that, in this situation, the brain expands while the head rocks anteriorly (Fig. 8C).

## Discussion

I have performed an experiment of electric stimulation of the head which has generated an increase in tension forces in the embryo, as a whole. The increase in tension causes a set of correlated movements. First we observe a shrinkage of brain vesicles, which is easy to explain: whenever there is tension in a shell, there exists a force oriented centripetally, which is opposed by the internal pressure. When increasing tension, the radius naturally shrinks. But there exist also belts of cells localized at the valleys separating brain vesicles, and along the midline (they actually are the very cause of the existence of these valleys). The belts of cells constrict too, thus adding to the shrinkage of the brain vesicle a linear stress exerted in the furrows separating brain vesicles (there also exists a similar sagittal furrow). Since the belts of cells constrict, they cause a stress oriented tangentially along the dorsal axis. This flexes the head in the dorsal direction. This explains why the head flexes in a retrograde movement, while the brain shrinks after the electric stimulation. Conversely, electrical stimulation is also able to evidence the correlation between head dilation and flexure, in the physiological sense of rotation. Indeed, electric stimulation causes also an increase in heart rate which induces brain dilation, and, simultaneously, an anterograde head flexure. This effect is easy to interpret: increase in pressure inside the brain shell itself (not inside the bulk, but really inside the curved plate of the vesicle) causes physically a decrease in in-plane tensile stress. This effect is strong enough to antagonize the contraction and cause an anterograde flexure, until the heart beat weakens, and a retrograde flexure occurs again (Video 11). It is important to understand that the vascular pattern mirrors the embryonic texture. In the tissue, stress is concentrated along the intervesicle valleys where belts of cells exert an anisotropic tensile stress; similarly, the main vessels follow the same cue, and these larger vessels also generate a more anisotropic and stronger tensile stress than capillaries.

Therefore, in both directions: brain dilation or contraction, brain deformation correlates with a built-in anterograde (resp. retrograde), flexure, at moderate values of stimulation (< 0.2). For higher values of the potentials (> 0.3 V) all effects mix up with, less often (N = 1/6), a massive retrograde flexure and brain contraction (Video 18). We ascribe the decrease in frequency of the retrograde effect at higher voltages to the kinetics of propagation of the contraction waves across the texture of contractile lines and main vessels, and to the increase of the sensitivity of the heart to electric shocks.

The observations and experiments reported here explain the most important “mysterious correlation of growth”, between brain dilation and head flexure. A dual physics exists, one for shell dilation one for flexure, related to 2D shell expansion, in the one hand, and 1D tension in the other hand. But the vesicles (the shells) can only exist if there are cables or belts of cells between them. Please note that the presence of the belts of cells patterns the neural tube in vesicles in close contact (with clefts occupying up to 2/3 of the lumen width). The mechanical interaction (or simply “the push”) between vesicles is the cause of the bending during dilation. Indeed, the numerical simulation finds that a *single* vesicle which balloons out does *not* bend.

Our main finding is therefore that a single vesicle does not flex, while a chain of vesicles flexes. In fact, a chain of vesicles (for example our 10-vesicles case above) can be viewed as a rod which undergoes internal compressive stress. If there were quite many cell belts, the vesicles could not balloon out and the problem would be similar to rod bending, without dilation. When there is a single vesicle, the problem is that of a dilation, but without bending. In effect, being a chain of vesicles, the brain is a physical object intermediary between a bubble and a rod, it has conserved properties of both: it dilates *and* it bends. Actually, it is not truly that brain dilation and head flexure are “correlated”, as if there were some hidden variable (for example some remote common genetic pathway): being a hollow tube with collars around it, the early brain is such a hybrid object that it intrinsically *curls* when it *dilates*, by physical visco-elastic constitutive law.

Of course, the experiments reported here show a transient effect, and we did not pursue development until hatching, for technical and ethical reasons. The purpose here was to evidence the existence of this internal correlation.

I acknowledge that the experiment was performed on chickens, and not on humans; needless to say, experiment on humans is forbidden by law. One may think of performing this experiment with other embryonic forms of vertebrates, for example mouse or fish embryos. One difficulty with mouse embryos is that it is quite complex to keep them alive after withdrawal from the uterus. Fish brains, on the other hand, do not balloon very much with respect to the questions discussed here. This is why the chicken model, which is classical in embryology, appears as suitable for these studies.

From a modelling point of view, an allometric relationship is found numerically between brain dilation and flexure. The simulation clearly shows that the dilation of the vesicles depends on their order, which may be one explanation for the fact that the nasal vesicle, the forebrain and midbrain are more developed than the hindbrain, at this stage. It is also worth noting that the phenomenon accelerates as flexure increases, with two distinct régimes which have a biological significance pointing to non-linear transitions between tubular brains and more flexed and dilated brains.

An important question for future studies is how the movements occurring at these early stages are upscaled in the final animal. Stated otherwise, do completely different and independent growth processes stack and capitalize on the early stages, or does the physics described here still play a role as the embryo grows and differentiates more. We think in particular of the role of neural crest cells in shaping the skull^29^. Also, when the brain folds, a more convoluted pattern of fibers is laid in cascade, still the original pattern of cell cables may be imprinted and play a role. During later stages, there exist developmental phenomena at larger scales, such as differential growth of the brain in birds, or the elongation of the jaws in cetaceans. It would be interesting to study how these modify the proportions of the early layout. For example, analysis of maxillary and mandibular extension in dolphins, which occur very late, still show a correlation with a retrograde rotation of the head (see for example Fig. 1 in Ref.^30^, and also a Video on Youtube^31^). Such movements might also occur during bird head growth. The upscaling of the early stages is also important to address with respect to the question of neoteny^32,33^. Juvenile traits in one species may correspond to adult traits in another “more advanced” species, while the traits in the juvenile form of that adult will correspond to a latent species; how this is biomechanically possible has been discussed in Ref. 23.

While the experiment of electric stimulation reported here may seem brutal in that it causes an ill controlled increase in tension in the entire head, it should be noted that evolution itself is not a well controlled experiment either, and that genetic mutations too cause global effects by changing parameters everywhere. As a matter of fact, it is remarkable that such a random assay as giving an electric shock to an embryonic head, induces so easily a well defined set of movements closely similar to what is reported in paleontology: the dynamic pattern which is elicited by external means is in fact already latent, built in the sample; the pattern of movement is controlled by the physics of it. The mechanism by which electricity causes increased tension has been investigated since the pioneering work of the Galvanis^34^. It is well known nowadays that actin-myosin contraction is dependent on potential because calcium channels are voltage dependent gates^35^. Conversely growth in itself causes a reduction in tension^12^ by cell-division pressure.

Electrical stimulation was used, here, to enhance tension forces and trigger contractions. However, it is not meant that electricity is directly the cause of evolution in craniates. Following the work of Levine et al.^36^, electric potentials in themselves are important cues which carry morphogenetic information, and regulate both metabolism and transcription pathways. Here, we only used electricity as an instrument to change forces by art. During evolution, forces can be changed otherwise, by mutations at different levels, either by changing the pathways for ionic concentrations, or by changing the interaction between actin filaments and myosin heads, or any other actor in the force actuation. We don’t mean here that evolution has played with electricity for humanization. Still, whether evolution did occur by a progressive change in potential set-points, within a built-in biaxial prepattern of contractions common to vertebrates, is a possibility, which we leave for further investigations.

The experiments reported here find also an important role for hemodynamics in the rotatory movements of the head during brain dilation. It is natural that mechanical features propagate from one compartment of the embryo to the others, by stress continuity across the tissue, which behaves as an effective average material. In the experiments we observe a dilation of the brain vesicles (perfused by capillaries), and an increased flow in the main vessels. Since these follow the embryo texture, the effect is a torque exerted by the dilating vesicles and the increased shear flow, which flexes the head more. Both are due to the same hemodynamic force. Thus the morphological correlation is by the texture. In general terms, in the absence of blood flow, all organs remain minute (diffusion alone does not permit animals to get bigger than a few millimeters). Heart pumping and blood flow allow animals to enlarge by a factor of up to × 1000. In this respect, hemodynamics enlarges all parts of the body, be it limbs, head or liver in the same way: blood flow is not in itself morphogenetic. However, in each organ, the texture drives the actual effect of the increased flow. In the head, the texture favors brain rotation, when the flow is increased, while the head gets bigger (Figs. 6D and 8C). This gives also support to previous studies of the correlation between head flexure and heart development^12,37^.

Careful observation of embryos after preparation shows that mechanical stimulation suffices also to influence head rotation and dilation.

In summary, electrical stimulation reveals the existence of an internal, built-in, correlation between brain dilation and curling of the head, deeply rooted in the fact that the brain is a hybrid physical object intermediary between a balloon and a rod. This provides experimental and theoretical evidence supporting the *Inside story* scenario of human origin: the fact that the head curls forward and the mouth recedes, as the brain dilates is, truly speaking, wired inside the brain texture by actual cell wires.

## Materials and methods

### Embryos preparation

Embryos are obtained from EARL Les Bruyères, Dangers 28100, France. The strain is ISABROWN strain from Hendrix genetics. Fertilized eggs are stored in a refrigerator at 14 °C, and used prior to 14 days of storage. Embryos are put to incubate during day zero, and observed on day 3. Generally, two batches of eggs are put to incubate, one around 10 AM d0, and one around 5–6 PM d0, to get embryos at the proper stage for observation in the morning and afternoon of d3. During the third day the embryos undergo a progressive transformation with an increase flexure and brain dilation (Video 1), during the same period of time the eye invaginates, embryos are best suited for observation after eye invagination, which perturbs brain dilation. The embryos are prepared for the experiment in the following way (Supp. Material, Figs. 1–5). Eggs are broken in a plastic cup (Supp. Mat. Fig. 1a). The embryo and the yolk sac are cut off from the vitelline membrane with fine scissors (Supp. Mat. Fig. 1b,c). The embryo + yolk sac are transferred to a Petri dish full of PBS (Dulbbeco) (Supp. Mat. Fig. 1d,e). The yolk and albumin are rinsed away (Supp. Mat. Fig. 1f). The embryo is transferred with a spoon to a glass disk with a removable aluminum ring serving as provisory dish (Supp. Mat. Fig. 1g,h). The vitelline membrane is removed with fine tweezers (Supp. Mat. Fig. 2a,b, arrow in (a,b) point to the vitelline membrane). The amniotic sac is torn apart very delicately with tweezers (Supp. Mat. Fig. 2c,d) in order to expose the head fully (Supp. Mat. Fig. 2e,f, red arrows point to the edge of the amniotic sac, which has to be removed). This step is critical, not only because observation across the amniotic sac is imperfect, but also because the amniotic sac itself is contractile, and leaving the head inside it would perturb the electric stimulation experiment. Then, the embryo is prepared in three important steps: first it is turned upside down, then the yolk sac is folded along the medio-lateral axis from top to bottom (Supp. Mat. Fig. 3a,b), and then it is folded along the antero-posterior axis twice: left–right, and right-left (Supp. Mat. Fig. 3c,d). By so doing the head appears now on a dark background, separated from the yolk sac. The aluminum ring is removed (Supp. Mat. Fig. 4a–c). The embryo on the glass disk is transferred under the microscope (for most experiments a Nikon Eclipse with long working distance objectives 4×, sometimes a Leica Macro Fluo). It is placed in the center of a shallow disk with a depth of PBS of approximately 3 mm (Supp. Mat. Fig. 4d). A heating stage with a round opening of 35 mm is positioned on top (from minitüb gmbh) (Supp. Mat. Fig. 4e). The midbrain is positioned such as to approach electrodes along the X axis of the microscope stage. The hole in the heating stage is closed with a glass disk, and a copper plate with a window (to avoid condensation, Supp. Mat. Fig. 4e,f). The copper plate is important in order to homogenize the temperature, and avoid condensation on the glass window. The embryo is left to recover for 15–60 min, until all spurious movements and capillary flows are quiescent. In particular, following mechanical stimulation during dissection, the embryo may spontaneously undergo a contraction of the head accompanied by a retrograde flexure, without any external electrical stimulation (Video 19). All these movements are left to relax prior to starting electrical stimulation. Heartbeat is monitored by video.

### Electrical stimulation

For the stimulation experiment, electrodes (Phymep, wolfram/Tu paired electrodes, 500 µm interdistance, coated with an insulator all the way down to approximately 20 µm from the tip) are attached to a thin pole, itself attached to a precision translation stage (Newport, Supp. Mat. Fig. 5b) The electrodes are oriented at 45° in order to be able to approach the head of the embryo below the objective (Supp. Mat. Fig. 5a–d show the whole set up). For the electric stimulation, the hole in the heating stage is reopened (Fig. 5c). The electrodes are descended in the hole (Supp. Mat. Fig. 5d white arrow) with a precision labo-lift or z-stage. The electrodes are approached almost in contact of the head along the median axis, in the area of the midbrain (Fig. 4A). The approach is monitored by video. A small electric pulse of amplitude 20–100 mV and duration generally 1 s is applied (with a pulse generator from AM systems Model 2100). Then the electrode is rapidly removed (< 3 s), and the hole is closed again. Most time-lapse videos are acquired with an acquisition rate of 1 frame/10 s. Closure of the hole in the heating stage is done manually, and it takes less than 10 s. The embryo is filmed with a CMOS camera from Basler (Phase gmbh), interfaced with ImageJ. Data is analyzed with custom macros (available by the author upon request).

### Particle imaging velocimetry

The Particle Imaging Velocimetry maps are done using the Tracker Module developed by Olivier Cardoso and Bérengère Abou. They are available upon request. A custom macro allows one to select by the mouse a box in which a grid of points is laid. The reference points are used for motion detection by correlation function between plates. A segment is drawn from the reference point, of length proportional to the movement.

### Video cardiography

Video Cardiography is performed using the “Stack Measure” plugin in ImageJ. “Stack Measure” measures the average “white” level in a region of interest (ROI) selected by the operator with the mouse. This level of white varies in a video of a beating heart (Supp. Mat. Fig. 7). If one selects a ROI in which one clearer part of the heart comes in and out during the beat, then the kinetics of heart wall can be measured. A video of the beating heart is generated with the highest possible frame rate, and highest possible contrast. Here 60 images/s. A ROI is selected close to the heart wall (Supp. Fig. 7). The heart wall diffuses light and appears whiter than its background. This contrast is used to measure the movement of the heart wall. During the heartbeat, as explained, the white feature of the heart wall will come in and out of the ROI, thus generating a signal which varies with a speed depending on the actual heart stroke. The more rapid the movement of the heart wall, the stronger the stroke. The “Stack Measure” plugin measures the average color value (in gray scale) of the box through the movie of the heartbeat. The temporal data are saved in a data file which is analyzed in KaleidaGraph. The mean color is plotted as a function of time, which gives the dynamics of the heart wall during time. A sharper rise corresponds to a more rapid movement of the heart wall, hence to a stronger stroke since the speed of the wall is the actual fluid speed at the exit of the heart (this is at variance with ECG, in which the potential does not measure directly the heart thrust).

### Blood vessels imaging

The density of erythrocytes is scarce at early developmental stages, and the blood vessels are quite transparent. In order to image properly the blood vessels, we enhance the images by integration of the blood flow over 200–600 plates in a video of the blood stream. However, the embryo moves (sometimes a lot) so we need to register the frames first. We have developed a custom macro to register the frames prior to extracting the blood vessels by the Z-stack (min) function in ImageJ. This produces a projection of the darkest erythrocytes, and the result is the formation of a composite image forming a digital cast of the blood vessels, in vivo, without any staining^38^. Imaging of the sagittal vessels in vivo is particularly challenging. In vivo, the embryo lies on its side and it is impossible to visualize the blood vessels on top of the head. This is all the more difficult as the yolk sac tends to wrap the head, as the embryo sinks into the yolk. In order to perform this imaging, the following steps proved necessary. First the amniotic sac is delicately torn apart with very fine tweezers. Then, a volume of approx. 4 ml of PBS is poured exactly on top of the head with a Pasteur pipette. By so doing a small coelom of buffer is formed around the head, which will prevent the yolk sac from wrapping the embryo again, and it will also keep the embryo moisturized. Next a slab of glass is cut in a glass slide by cutting the glass slide in two along the long axis. This slab is attached to a mechanical lever or pole, itself mounted on a vertical precision translation stage (Newport). The edge of the glass slab is descended on top of the embryo body, along the neck, quite laterally, so that a torque is exerted very gently on the embryo neck. The precision stage is motorized (controller ESP300 from Newport), so that the speed of descent can be adjusted in the micrometer/s range. As pressure is exerted on the neck, the head rotates, and it can thus be positioned steadily in sagittal view. Then, a movie of the blood flow is generated and integrated following the procedure described in Ref.^38^, in order to get the enhanced images of Fig. 6D. Images were acquired at different orientations for the HH21 stage (Fig. 6D Right). This allows one to see in detail, in vivo, without any staining or fluorescent dye, the blood flow and the vessels. In particular, we evidence that the vascular texture follows the embryonic texture in that brain vesicles are covered with a fine plexus, while larger vessels follow the sagittal valley and the intervesicle valleys. Assuming that vessels exert a linear stress, it is inferred that the capillary plexus exerts a roughly homogeneous surface stress over the dilated brain neural ectoderm, while larger vessels exert a bigger but more concentrated linear stress, this serves as justification for the numerical calculation in Fig. 8C.

### Ethics declarations

All experiments are allowed under French law “code rural R214-87 to R214-137” which authorizes scientific experimentation on chicken embryos at all stages until hatching, without approval from an ethics committee. All experiments were carried out in accordance with existing guidelines and regulations. Animal experimentation are reported following ARRIVE guidelines.

### Supplementary Information

Supplementary Information 1.

Supplementary Figures.

Supplementary Information 2.

Supplementary Video 1.

Supplementary Video 2.

Supplementary Video 3.

Supplementary Video 4.

Supplementary Video 5.

Supplementary Video 6.

Supplementary Video 7.

Supplementary Video 8.

Supplementary Video 9.

Supplementary Video 10.

Supplementary Video 11.

Supplementary Video 12.

Supplementary Video 13.

Supplementary Video 14.

Supplementary Video 15.

Supplementary Video 16.

Supplementary Video 17.

Supplementary Video 18.

Supplementary Video 19.

## Data Availability

All data and codes are available upon request by the author Vincent Fleury Laboratoire MSC, 10 rue Alice Domont et Léonie Duquet 75013 Paris, France, vincent.fleury@u-paris.fr.
