# Supplementary material for: Electrical stimulation of chicken embryo development supports the Inside story scenario of human development and evolution
Source: Sci Rep. 2024 Mar 27;14:7250. doi: 10.1038/s41598-024-56686-y (PMC10973335; doi:10.1038/s41598-024-56686-y)
Supplement: Supplementary file 1 — Supplementary Information 1. [file 41598_2024_56686_MOESM1_ESM.pdf]

Supplementary Material Videos

Video 1. In vivo imaging of head flexure during chicken embryo development. During early stages of chicken development, the entire head flexes forward. This movement induces the formation of a neural tube in the form of a "archbishop's crosier" (Mag. 0.7X, day 2, duration 15hrs).

Video 2. Dorsal view of the embryo during the electric stimulation of the head showing contraction of the intervesicle belt of cells (large dashed line) and brain reduction, with the final result which is a retrograde rotation of the entire head (Mag. 10X, total time 60Min.). Please note that the sagittal valley constricts too (small dashed line).

Video 3. Profile view of the head during the electric stimulation, showing the retrograde rotation of the head in Fig. 3F Top (not the same embryo as Video 2, Mag 2X, total time 90Min.). bv: brain vesicle; hb: hindbrain; nv: nasal vesicle; H: heart; op: otic pit; map: mandibular primordium; e: eye.

Video 4. Simultaneous brain shrinkage and rotation. The video shows an animation between two plates separated by 10 minutes, in order to evidence the two types of movements.

Video 5. Effect of an electric stimulation on a wound (incision) in the brain. An embryo (day 3) is prepared and left to recover a few minutes in the incubator, then an incision is rapidly made with a scalpel. The incision is left to evolve for 5 minutes, after which electrodes are approached (one polarity on each side of the wound), and an electric stimulation is performed (0.15V, for 1 Sec.). The electric stimulation induces a more rapid and stronger opening of the wound. (Mag. 4X, total time 40Min.). e: eye; map: mandibular primordium; op: otic pit; H: Heart.

Video 6 Incision control experiment: incision without electric stimulation (to the left the global view, to the right a close up view of the wound). The wound forms an open cleft very rapidly (<10sec), the tissue reorganizes along the wound, it forms an elliptic ring which constricts and the cleft rapidly closes and heals (Mag. 4X, total time 1.5hour).

Video 7. Effect on vascular tone. Electric shocks, especially for higher values >0.1V may cause heart stimulation. This causes a vasodilation, quite visible here at Mag. 10X (total time 25Min.).

Video 8. Effect of heart acceleration on head rotation and flexure. When the heart tone increases, the brain dilates and the head flexes anteriorly. Blood vessels dilate. (Mag. 4X, total time 53Min.). bv: brain vesicle; cv: cardinal vein; nv: nasal vesicle hb: hindbrain; map: mandibular primordium; e: eye.

Video 9. Other example of brain dilation and head flexure when the heart tone increases. (Mag. 4X, total time 50Min.). bv: brain vesicle; v: vein; e: eye.

Video 10. Concatenation of 2 images showing the simultaneous brain dilation and head flexure, the arrowhead points to the dilating vein. Mag. 4X.

Video 11. When the heart starts to weaken, the head constricts again and the head rocks in a retrograde mode. (Mag. 4X, total time 115Min.).

Video 12. Retrograde movement and brain shrinkage observed when the omphalomesenteric artery is poked, and the embryo bleeds. As the embryo bleeds, the brain constricts and the head flexes backwards. (Mag. 4X, total time 23Min.).

Video 13. Numerical simulation of a single vesicle dilation, with a given internal pressure. One observes a mere dilation of the vesicle, without downwards nor upwards flexure.

Video 14. Numerical simulation of a single vesicle expansion, with an additional tension opposing dilation, and causing a posterior shear force in between vesicles. One observes an uplift or retrograde flexure of the vesicle.

Video 15. Numerical simulation of a line of four vesicles expanding under pressure. One observes a downwards flexure of the line of vesicles. The 4 vesicles case corresponds to the physiological one. The dynamics is to be compared to the one in Video 1.

Video 16. Numerical simulation of a line of ten vesicles expanding under pressure. This movie was used for the graphs in Fig. 7D. The acceleration of the flexure and of vesicle dilation is quite visible.

Video 17. Numerical simulation of a line of five vesicles, dilating with a given internal pressure, with an additional tension opposing dilation, and causing a posterior shear force in between vesicles. The line of vesicle is straightened, and it shows a retrograde rotation, with respect to the case with a smaller tension.

Video 18. Massive retrograde rotation and head constriction for a value of the potential =0.3V. (Mag. 4X, total time 48Min.)

Video 19. Example of a spontaneous retrograde rotation and head flexure, observed after embryo preparation, without any electric stimulation. These movements are due to mechanical stimulation during manipulation of the embryo (Mag. 4X, total time 90Min.)
