# Supplementary material for: Electrical stimulation of chicken embryo development supports the Inside story scenario of human development and evolution
Source: Sci Rep. 2024 Mar 27;14:7250. doi: 10.1038/s41598-024-56686-y (PMC10973335; doi:10.1038/s41598-024-56686-y)
Supplement: Supplementary file 2 — Supplementary Figures. [file 41598_2024_56686_MOESM2_ESM.pdf]

Supplementary Material Figures

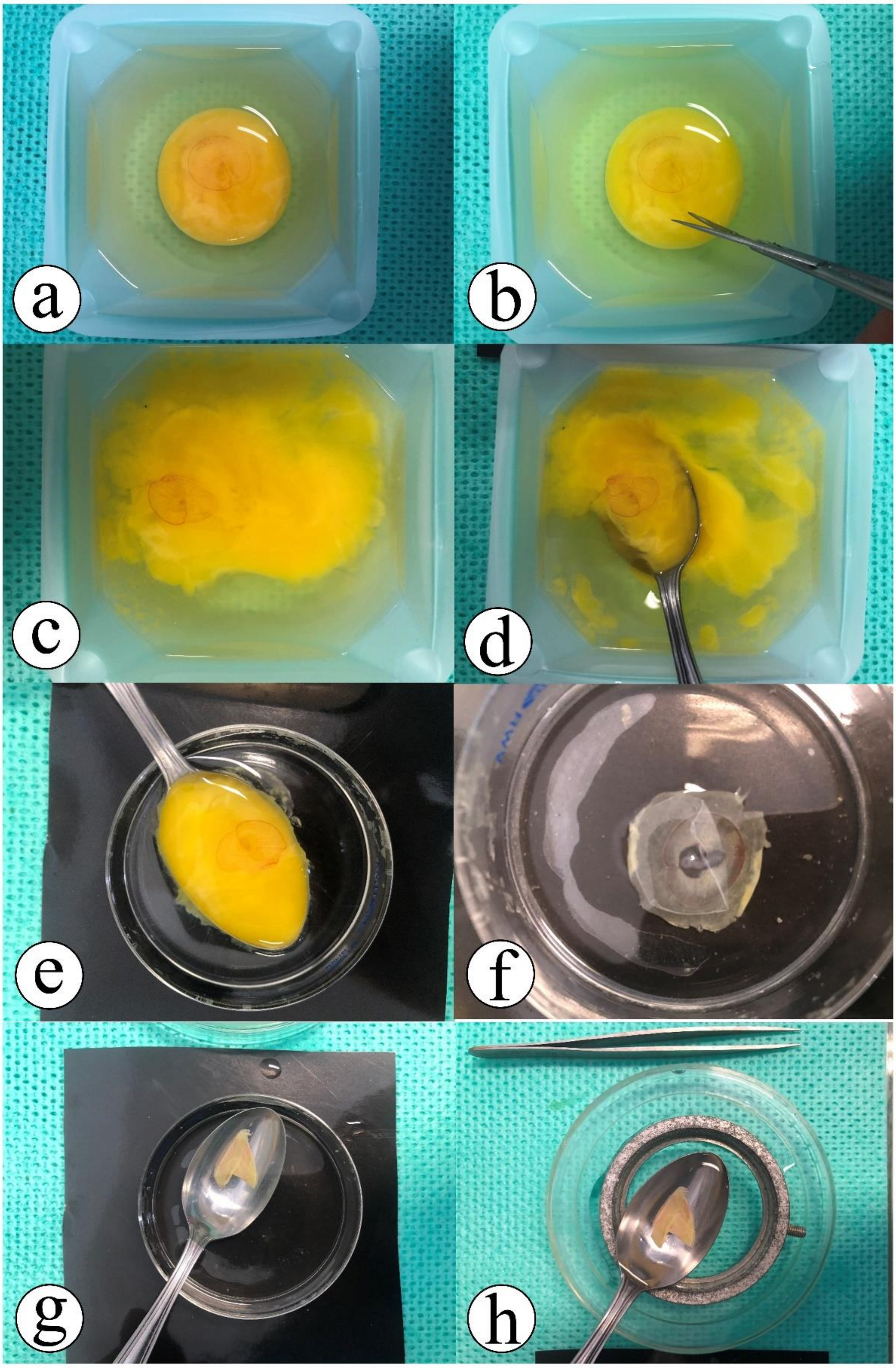

Supplementary Figure 1

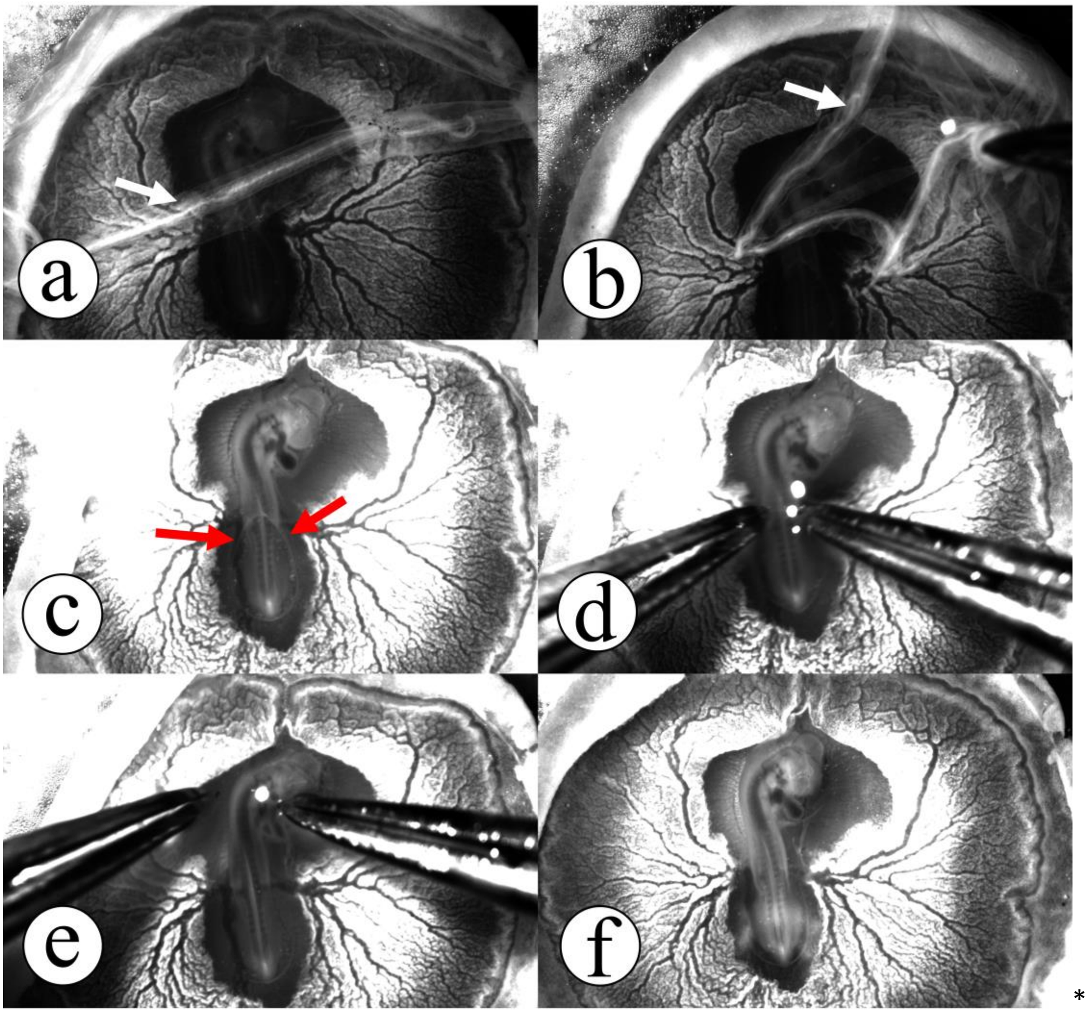

Supplementary Figure 2

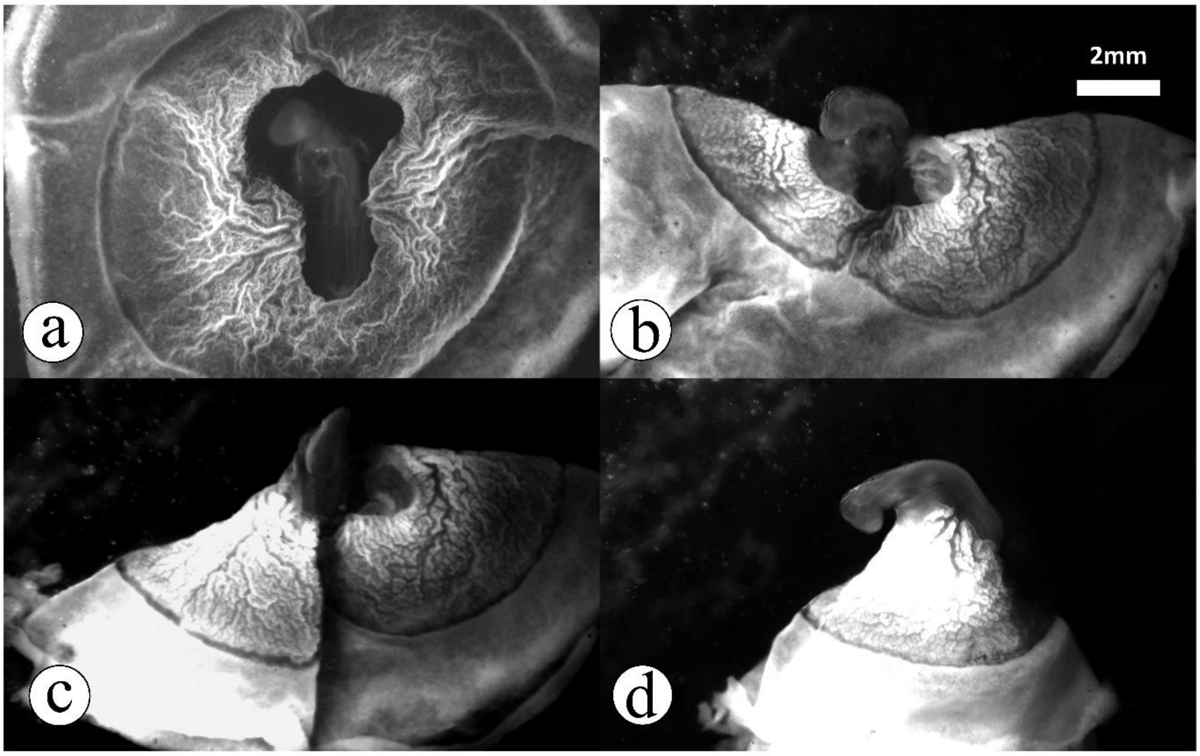

Supplementary Figure 3

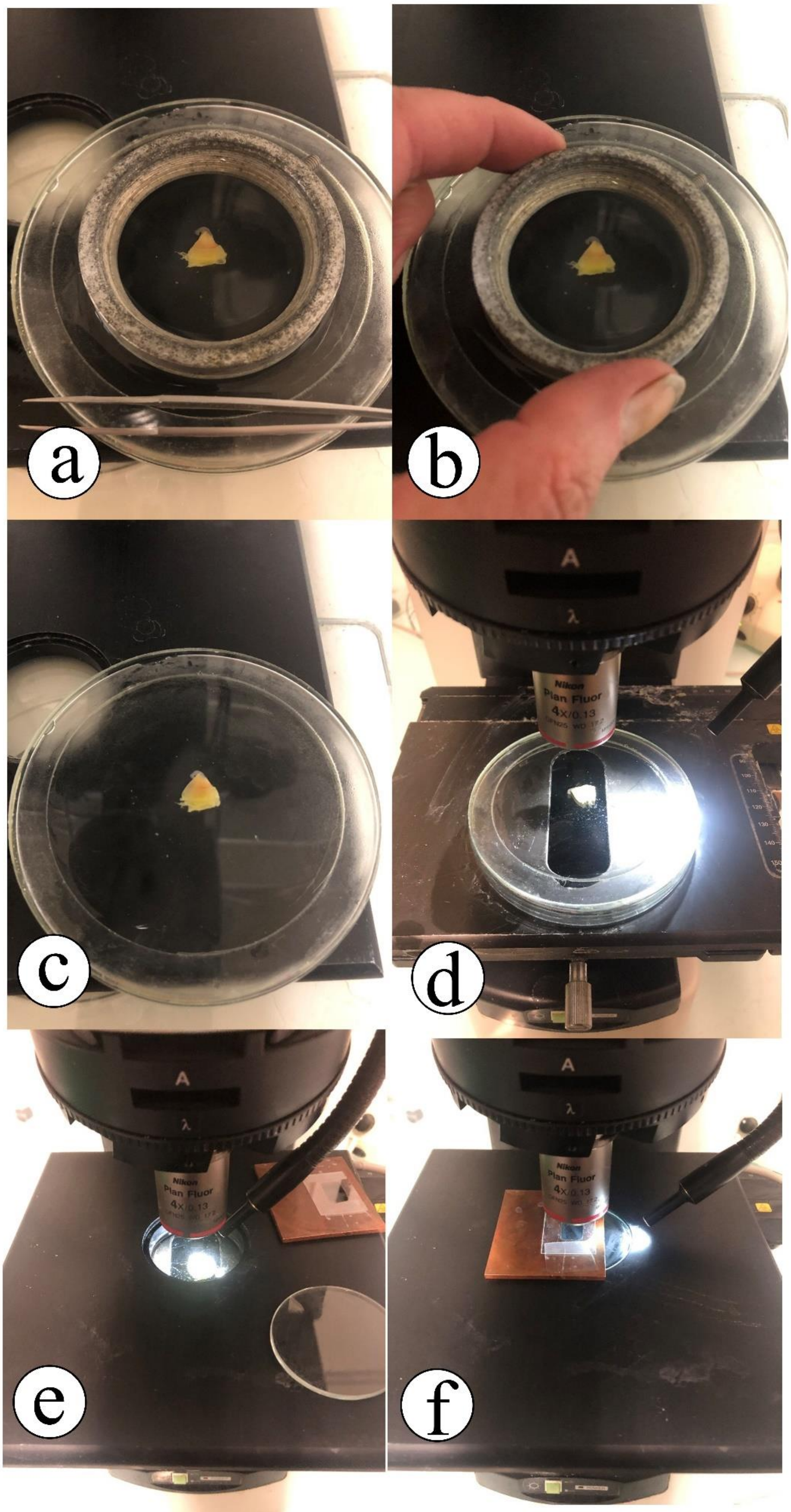

Supplementary Figure 4

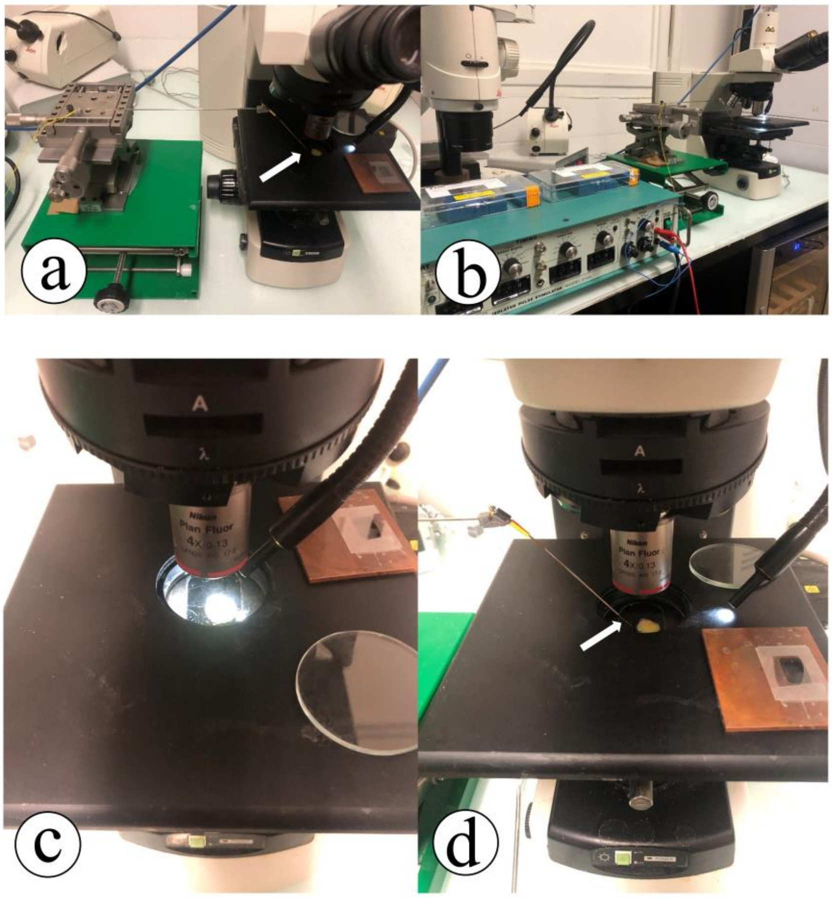

Supplementary Figure 5

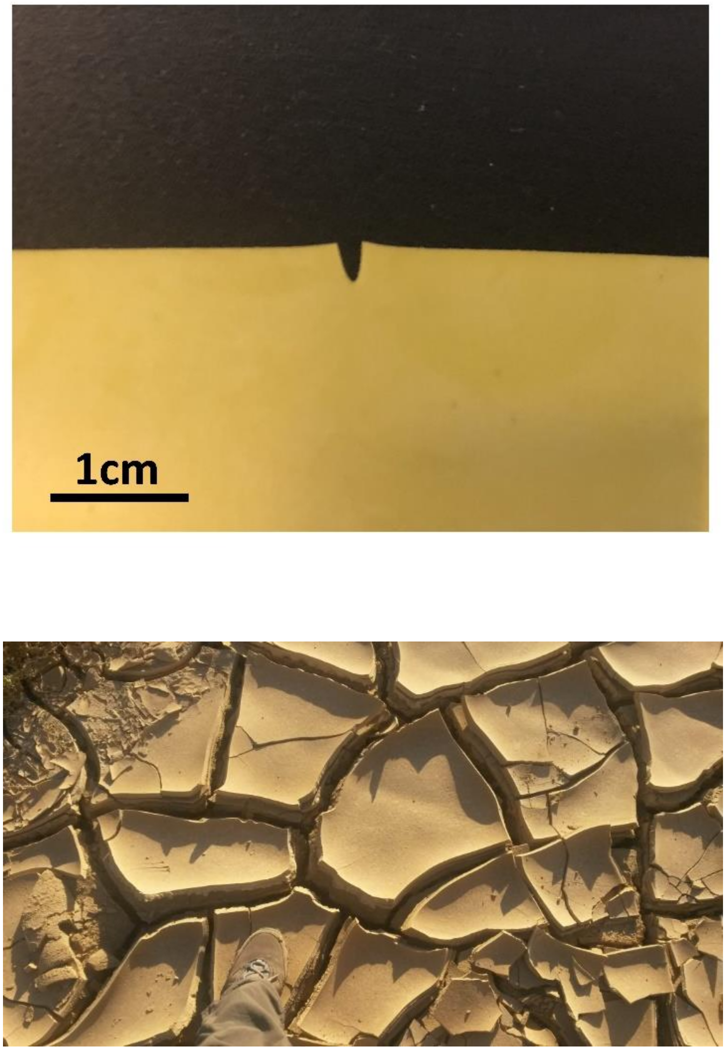

Supplementary Figure 6

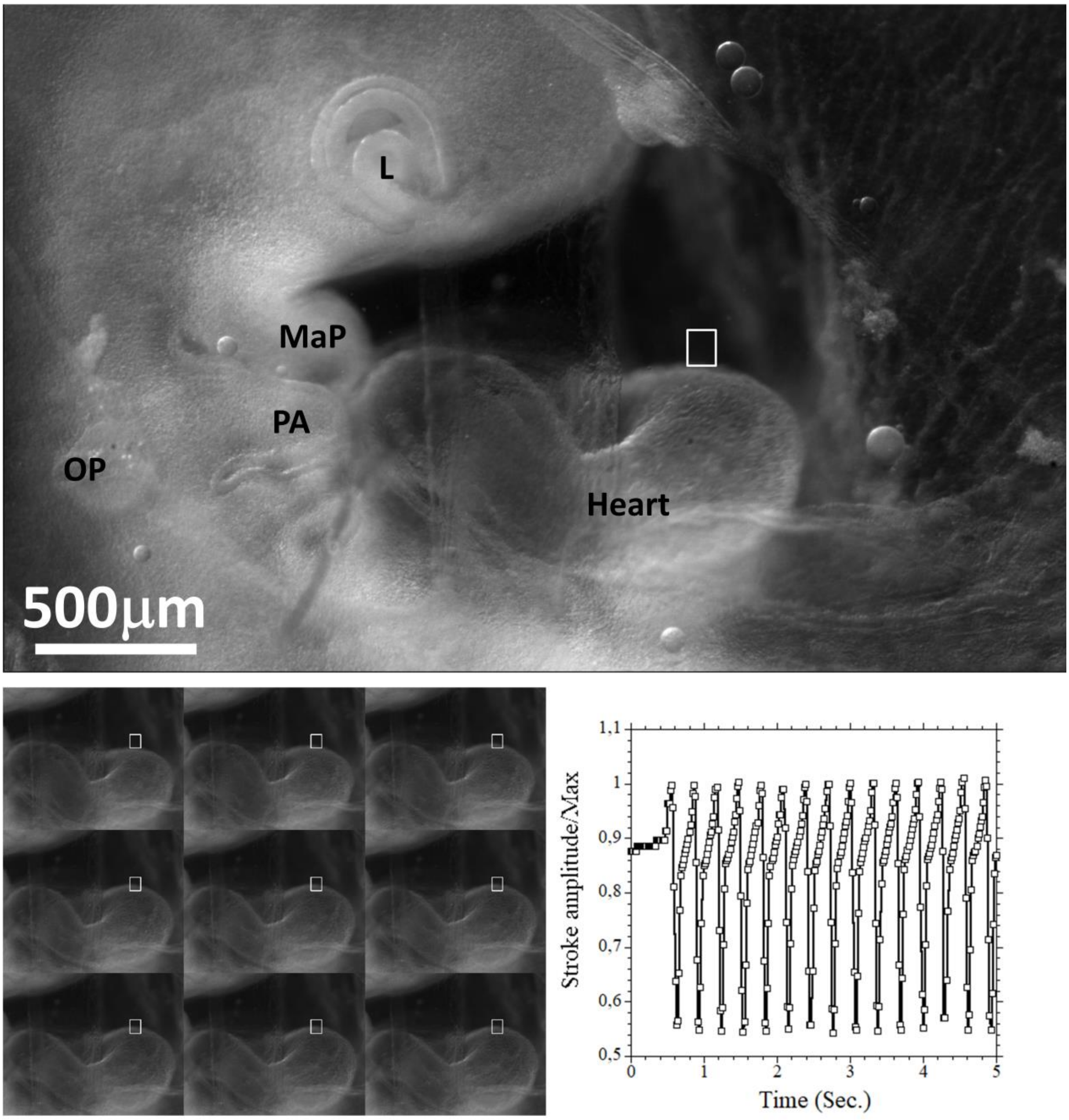

Supplementary Figure 7

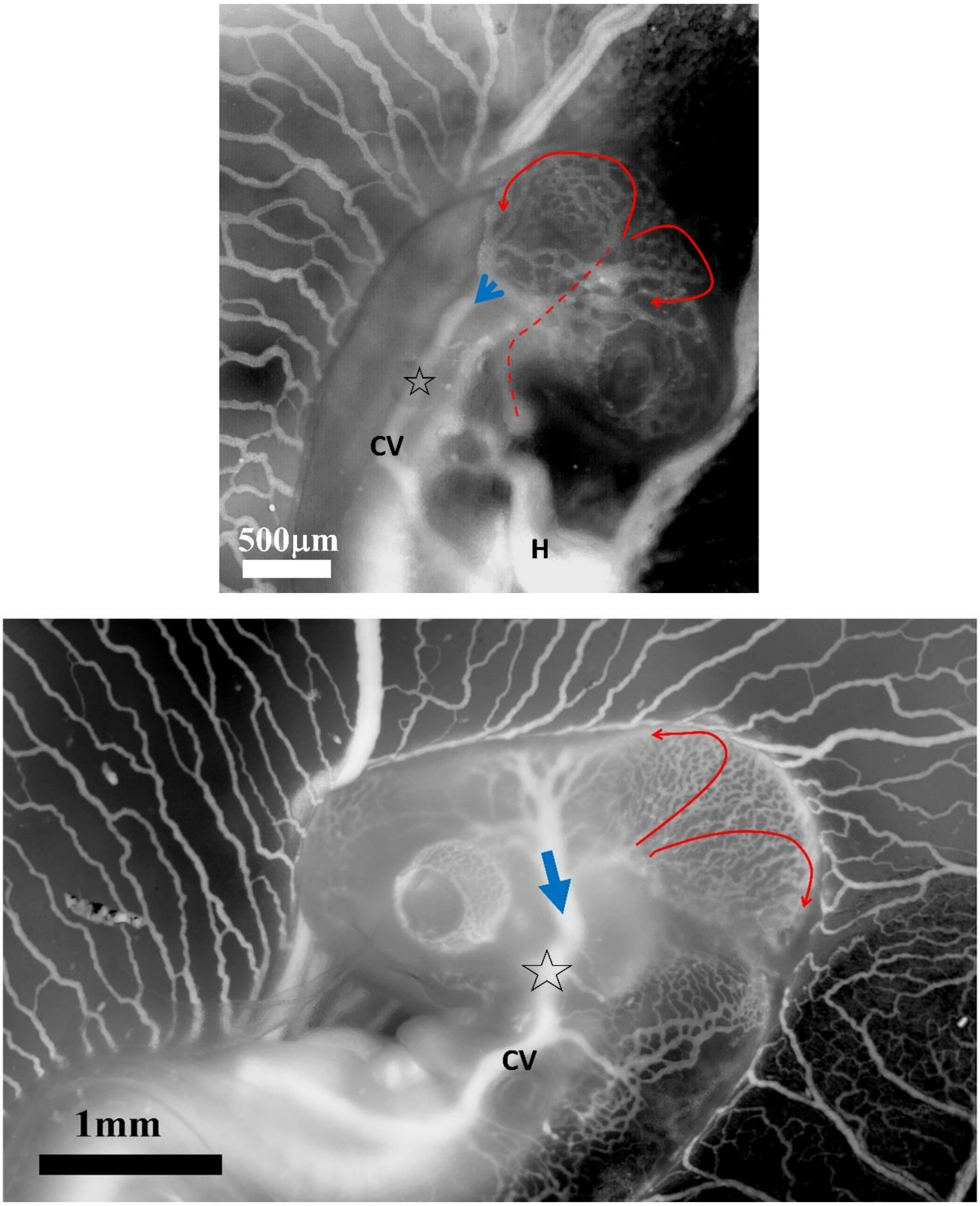

Supplementary Figure 8

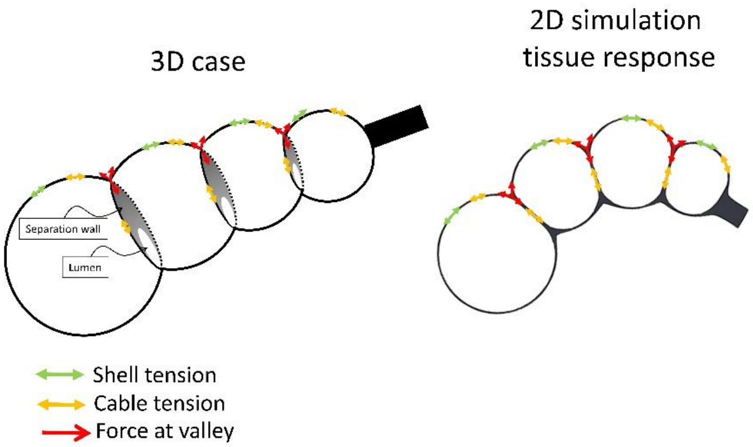

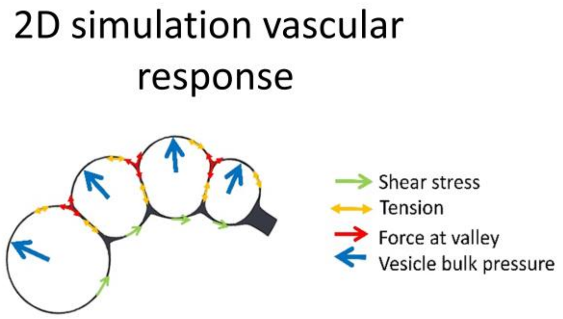

Supplementary Figure 9
