# Supplementary material for: Electrical stimulation of chicken embryo development supports the Inside story scenario of human development and evolution
Source: Sci Rep. 2024 Mar 27;14:7250. doi: 10.1038/s41598-024-56686-y (PMC10973335; doi:10.1038/s41598-024-56686-y)
Supplement: Supplementary file 3 — Supplementary Information 2. [file 41598_2024_56686_MOESM3_ESM.pdf]

Supplementary Material Figures Legends

Supplementary Figures 1 to 5 Embryo preparation Steps for embryo preparation and electric stimulation (see Materials and methods).

Supplementary Figures 6 Mechanical analogs of wound formation The classic form of a wound is obtained by simply incising the edge of a stretched rubber foil[25] (Supp. Fig. 6 Top). The lip of the wound spontaneously bends outwardly due to the gradient of stress between the stretched material and the edge of the wound (where the stress vanishes). Similar outward bending is casually observed in mud cracking under desiccation by stress relief at the crack edge (Supp. Fig. 6 Bottom, photo courtesy of Evelyne Mervine).

Supplementary Figures 7 Video Cardiography (see Materials and methods). OP: Otic Pit; MaP: Mandibular Primordium; L: Lens; PA: Pharingeal Arches.

Supplementary Figure 8 Pattern of blood vessels and flow direction In vivo low resolution view of the blood vessels obtained by integration of the blood flow in the head at HH19 and HH21 (see Materials and methods, or Ref. 36). The arrows show the flow direction. Arterial flow enters into the capillary plexus from the arteries which are located deeper inside the embryo, while it returns via the more superficial cardinal veins. The cardinal veins are labelled CV and indicated by a star. This in vivo imaging shows that the cardinal veins follow exactly the embryo texture, below the neural tube. H: Heart.

Supplementary Figure 9 Correspondence between the 3D ideal situation and the 2D model In an ideal 3D modelling of the neural tube (Left), the tensions in the ring (yellow) induce a resulting centripetal force (red). These forces are fought by the tension in the shell (green), which result in a force along the ring (red). In the real situation, the separation wall between vesicles is about 2/3 of the lumen. In the 2D simplified model (right), the situations for the forces and tensions are equivalent. But in order to have a tension force associated to wall elongation, the column has to span the entire lumen width instead of the 2/3. While the model might be partially erroneous at start, it fares better as the brain vesicles dilate more and the cleft deepens. Along the sagittal line (median axis), runs also a wire which carries a tension. The top right scheme shows the hypothesis used for the simulation of the effect of increased tension in the tissue (increase in stress in the vesicle, and posterior shear in the upper boundary). Conversely, the main blood vessels exert tension along the lower boundary, and dilation in the brain plexus, this gives a scheme of the stresses as in the bottom image.
